# *Clonostachys rosea* ‘omics profiling: identification of putative metabolite-gene associations mediating its in vitro antagonism against *Fusarium graminearum*

**DOI:** 10.1186/s12864-023-09463-6

**Published:** 2023-06-26

**Authors:** Adilah Bahadoor, Kelly A. Robinson, Michele C. Loewen, Zerihun A. Demissie

**Affiliations:** 1 Metrology Research Center, National Research Council Canada, 1200 Montreal Rd, Ottawa, ON K1A 0R6 Canada; 2 Aquatic and Crop Resource Development, National Research Council of Canada, Ottawa, ON Canada

**Keywords:** Beneficial fungi, *Clonostachys rosea*, Data integration, Gene clusters, IntLIM, Epidithiodiketopiperazine, Specialized metabolites

## Abstract

**Background:**

*Clonostachys rosea* is an established biocontrol agent. Selected strains have either mycoparasitic activity against known pathogens (e.g. *Fusarium* species) and/or plant growth promoting activity on various crops. Here we report outcomes from a comparative ‘omics analysis leveraging a temporal variation in the in vitro antagonistic activities of *C. rosea* strains ACM941 and 88–710, toward understanding the molecular mechanisms underpinning mycoparasitism.

**Results:**

Transcriptomic data highlighted specialized metabolism and membrane transport related genes as being significantly upregulated in ACM941 compared to 88–710 at a time point when the ACM941 strain had higher in vitro antagonistic activity than 88–710. In addition, high molecular weight specialized metabolites were differentially secreted by ACM941, with accumulation patterns of some metabolites matching the growth inhibition differences displayed by the exometabolites of the two strains. In an attempt to identify statistically relevant relationships between upregulated genes and differentially secreted metabolites, transcript and metabolomic abundance data were associated using IntLIM (Integration through Linear Modeling). Of several testable candidate associations, a putative *C. rosea* epidithiodiketopiperazine (ETP) gene cluster was identified as a prime candidate based on both co-regulation analysis and transcriptomic-metabolomic data association.

**Conclusions:**

Although remaining to be validated functionally, these results suggest that a data integration approach may be useful for identification of potential biomarkers underlying functional divergence in *C. rosea* strains.

**Supplementary Information:**

The online version contains supplementary material available at 10.1186/s12864-023-09463-6.

## Background

*Clonostachys rosea* is an endophytic filamentous fungi recognized for its microparasitic and plant growth promotion (PGP) properties. Subsequently, a few *C. rosea* strains have been developed into biocontrol and/or biostimulant agents against economically important plant pathogens. Two strains of *C. rosea*, ACM941 and 88–710, have been patented for different applications. ACM941, originally isolated from pea plants was patented for commercial use in Canada as a biocontrol agent against *Fusarium graminearum* [1, 2]. *F. graminearum* is a fungal plant pathogen that mainly causes Fusarium head blight in North America. *C. rosea* strain 88–710 was patented in both the United States of America and Canada for its benefits in promoting plant vigor, health, growth and yield [3]. Despite the differences in their patented applications, the two strains are genetically closely related [4, 5] and their beneficial properties display strong overlap. *C. rosea* strain 88–710 is well regarded for its strong PGP ability but is also noted to induce disease resistance against economically important fungal pathogens in different crops including cereals, vegetables, peas, etc. [3]. On the other hand, ACM941 elicits superior mycoparasitism activity, but still has some PGP activity as well. Leveraging these beneficial activities could enhance the economic competitiveness of these biocontrol agents compared to chemical-based products. Toward this we have undertaken a systematic comparative ‘omics approach to glean insight into the molecular and biochemical underpinnings of the beneficial effects of *C. rosea*.

At this time, both direct and indirect (comparative genome analyses and ‘omics profiling) evidence has established specialized metabolites (SMs) as leading candidates mediating PGP and mycoparasitic properties of beneficial microbes. For example, harzianic and isoharzianic acids, isolated from *Trichoderma Harzianum* (also a microparasitic fungi), were shown to promote tomato growth and impart resistance against fungal pathogens like *Pythium irregulare*, *Sclerotinia sclerotiorum* and *Rhizoctonia solani* [6]. Likewise, SMs isolated from *C. rosea* strains ACM941 [7], IK726 [8], YRS-06 [9] and BAFC3874 [10] displayed promising antibacterial and antifungal activities. Additionally, we have recently shown that SMs of *C. rosea* strain ACM941 modulate early stage effects during *F. graminearum* mycoparasitism [11]. Such prior studies, each relying on independently analysed, single platform ‘omics profiling techniques, have yielded several beneficial lead metabolites and possible associated biosynthetic genes, including several putative gene cluster identifications [7, 9–13]. However, the vast majority of *C. rosea* SMs and their metabolic pathways remain to be characterized. For example, while deletion of a non-ribosomal peptide synthase gene 1 (*nrps1*), identified based on its expression profile, significantly weakened the nematicidal and mycoparasitic properties of *C. rosea* strain IK726 [8], any *NRPS1*-linked metabolite has yet to be identified.

Genomic-comparison is in itself a powerful tool for prediction of structural and functional features responsible for phenotypic differences between closely related organisms. For example, comparative genomics analysis was used to identify genetic features unique to the *E. coli* strain NADC6564 and to infer their roles in strain virulence [14]. However, recent advances in data integration tool development and availability of high-throughput genomic and metabolomic data have also significantly enhanced our ability to correlate functionally related transcripts and metabolites. In particular, transcriptomic and metabolomic data can be integrated using different approaches including multivariate data analyses, differential correlation/co-expression or pathway/network based analyses methods [15–25]. Integration of multiple types of high-throughput ‘omics profiling data could thus be used to elucidate global patterns that might explain mechanisms that drive *C. rosea’s* mycoparasitism, as well as reveal putative genes and metabolites involved in the process. To this effect, several open-source computational solutions have been developed to integrate metabolomics and transcriptomics data including MixOmics [20], WGCNA [17, 18], DiffCorr [15], MetaboAnalyst [19], INMEX [24], XCMS Online [16], Metabox [23], IMPaLA [26], IntLIM [27], etc.

However, while multivariate and correlation-based data integrations reveal global transcript and metabolite abundance patterns, they can’t identify direct transcript and metabolite associations. Alternatively, network/pathway-based approaches are an excellent tool to associate transcripts with their corresponding metabolites, but are limited to metabolites and transcripts that can be mapped to functionally characterized pathways, ultimately limiting their application to model organisms. In fact, only 16.3% of metabolites from the Human Metabolome Database have been detected and quantified, and few of them can be mapped to pathways [28]; and this number becomes negligible for non-model organisms like *C. rosea.* As such, applying pathway-based transcript and metabolomic data integration is impractical. However, IntLIM (Integration through Linear Modeling), a publicly available R package data integration tool, was specifically developed to identify novel direct relationships between gene and metabolite pairs potentially governing phenotypic- or genotypic- specific variations among biological samples. Favourably, the availability of a well curated genomic and metabolomic database isn’t a prerequisite to integrate multiple high-throughput data using IntLIM [27].

Consistent with their functional similarities, the genomes of the *C. rosea* strains ACM941 and 88–710 are highly conserved [4, 5]. Thus, the subtle differences in relative mycoparasitism and PGP activities between these strains are likely the result of fairly limited variation in their encoded genetic material, which in turn manifests as differential metabolite accumulation. In this current study, we tested the hypothesis that genetic and metabolic factors underlying the mycoparasitic property differences between strains ACM941 and 88–710 could be correlated using transcriptomic and metabolomic data integration tools developed for non-model organisms such as IntLIM. Here we report the outcomes of this investigation, highlighting metabolites and gene associations that could potentially serve as biomarkers for mycoparasitism.

## Results

### ***C. rosea*** ACM941 and 88–710 exometabolite fractions show temporal variations in growth inhibition activity against ***F. graminearum***

**Fig. 1:**
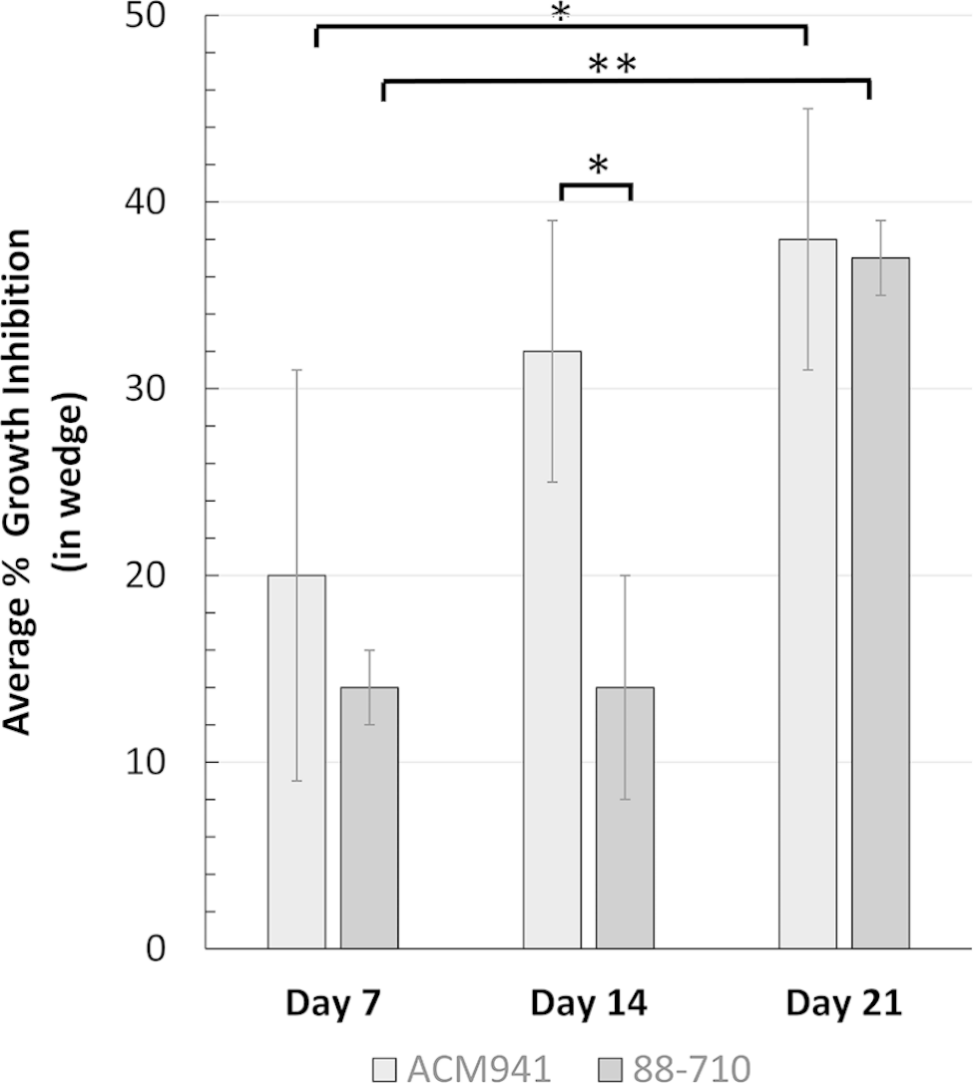
Growth inhibition of *F. graminearum* strain GZ3639 by exometabolites of *C. rosea* strain ACM941 (1) and 88–710 (2) fermented in Czapek Dox media for 7 days, 14 days and 21 days as indicated

Initially the biological activity of spent media exometabolite extracts from strains ACM941 and 88–710 were investigated. While similar *F. graminearum* growth inhibition levels were observed arising from exometabolites extracted from 7-day old cultures (Fig. 1 (also see Additional file 1: Fig. S1)), a significant increase in inhibition was observed for day-14 ACM941 extracts. This effect was maintained for day-21 ACM941 extracts. In contrast, while strain 88–710 extracts did eventually achieve increased *F. graminearum* growth inhibition, this only became detectable in extracts obtained from 21-day old cultures.

### Exometabolite accumulation profiles highlight temporal variations between ***C. rosea*** strains ACM941 and 88–710

**Fig. 2:**
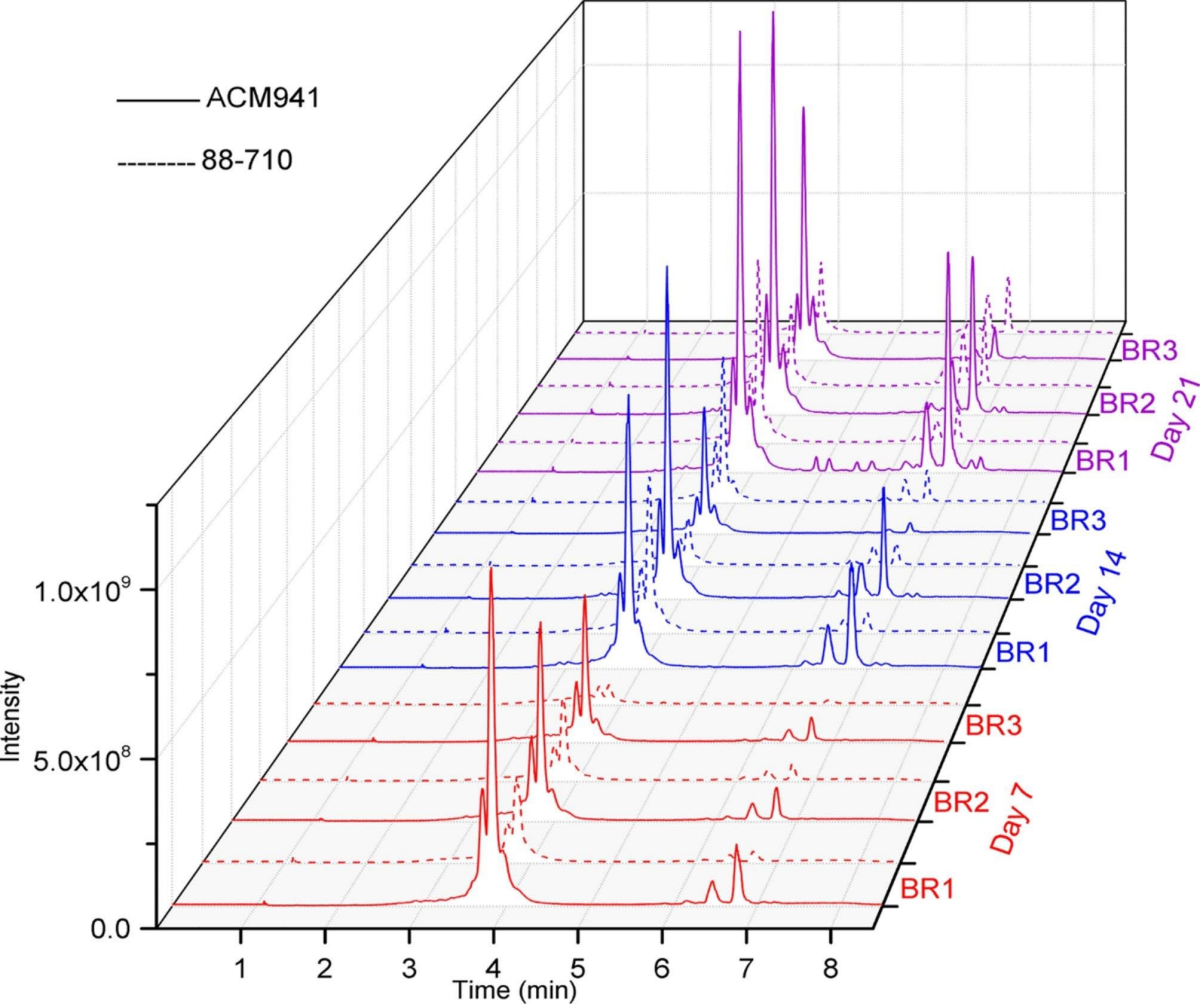
Differential exometabolic profiles of *C. rosea* strain ACM941 (solid lines) and 88–710 (dotted lines) after 7-, 14- and 21-days fermentation in Czapek Dox media, compared to unused medium. The LC-MS chromatogram of all three biological replicates (BR) at each time point are shown

In order to identify *C. rosea* metabolites whose accumulation patterns might match the observed *F. graminearum* growth inhibition trends, we used untargeted metabolomics profiling. Overall, both *C. rosea* strains (ACM941 and 88–710) secreted a core set of common metabolites, although the number of different metabolites detected and their accumulation levels showed significant differences depending on the strain and fermentation time period. In general, ACM941 was a more vigorous strain producing higher levels and a more diversified set of compounds than 88–710. After 7 days of culturing, the levels of detected exometabolites secreted by ACM941 were 2–3 fold higher than 88–710, and it maintained its higher production levels up to day 21 (Fig. 2 (solid lines)). With respect to strain 88–710, although metabolite accumulation was slower to start, levels increased by 21 days of culturing (Fig. 2 (dashed lines)).

Overall, two major groups of metabolites were observed from both strains. The group of metabolites eluting in the 3.5–4.5 min range were already present at high levels by day 7 and their production levels did not fluctuate much throughout the 21 day experiment. Any involvement of these metabolites in inhibiting *F. graminearum* growth was therefore deemed unlikely. On the other hand, the metabolites eluting in the 6–7 min range showed an accumulation level that appeared to correspond to the growth inhibition pattern observed in Fig. 1(also see Additional file 1: Fig. S2A&B). The clear inhibition activity of the day 14 ACM941 extract, and the delayed inhibition response of 88–710 to day 21 extracts matches the increased concentration of these metabolites in the culture media at these time-points (Additional file 1: Fig. S3A&B). Thus, it is possible that one or a subset of these exometabolites may be involved in *F. graminearum* growth inhibition.

**Fig. 3:**
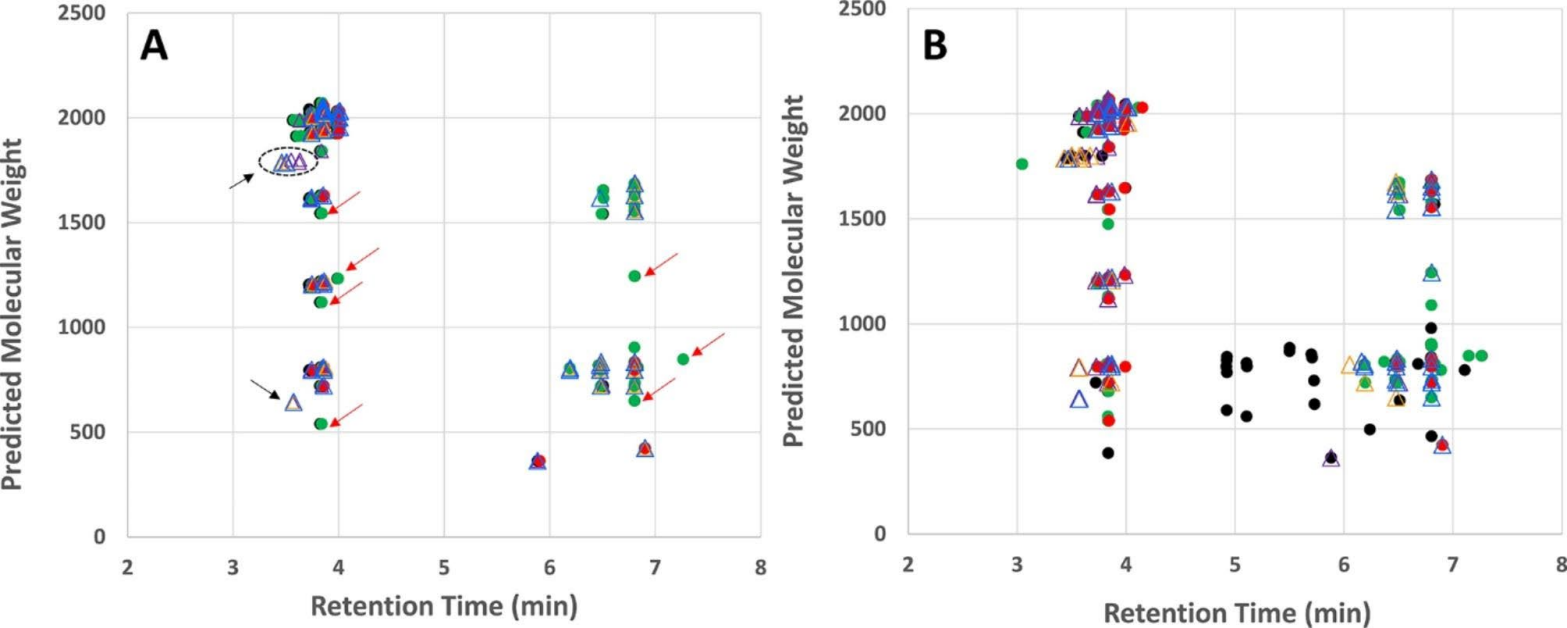
The detected secretomes of *C. rosea* strains ACM941 and 88–710. **(A)** Day 14 culture extracts. The secretome of strains ACM941 (filled circles with black (BR 1) green (BR 2) and red (BR 3)) and 88–710 (empty triangles, with purple (BR 1), orange (BR2) and blue (BR3)) have an overall similar profile, although some metabolites unique to ACM941 (red arrows) and 88–710 (black arrows) are detected. **(B)** Day 21 culture extracts. More diverse metabolites were detected for both strains, with ACM941 producing more unique metabolites than 88–710

The molecular weight range of the secreted metabolites together with gene cluster analyses, described below, provided a first step in predicting the types of compounds secreted by ACM941 and 88–710 (Fig. 3). The vast majority of the metabolites were high molecular weight compounds (> 500 amu). In particular, compounds detected in the molecular weight range 500–1000 amu were predicted to potentially belong to polyketides synthase (PKS) natural products, non-ribosomal peptides (NRPs) or PKS-NRP hybrids. Those exhibiting molecular weights > 1500 amu, typically produced doubly charged ions, a hallmark of larger peptides. It is interesting to note that in general, the larger peptide-like metabolites eluted early, in the 3.5–4.5 min range. These peptide-like metabolites were also secreted early in the culturing time, and thus did not match the biological activity of the extracts that had increased inhibitory properties at 21 days. Whereas the biological activities of ACM-941 and 88–710 were more clearly correlated with the accumulation of compounds that eluted later in the 6–7 min range and in the molecular weight range 500–1250 amu. It is therefore likely that the metabolite(s) of interest belonged to the PKS, NRP or PKS-NRP class of natural products.

### Gene expression profile of ***C. rosea*** strain ACM941 compared to strain 88–710

**Fig. 4:**
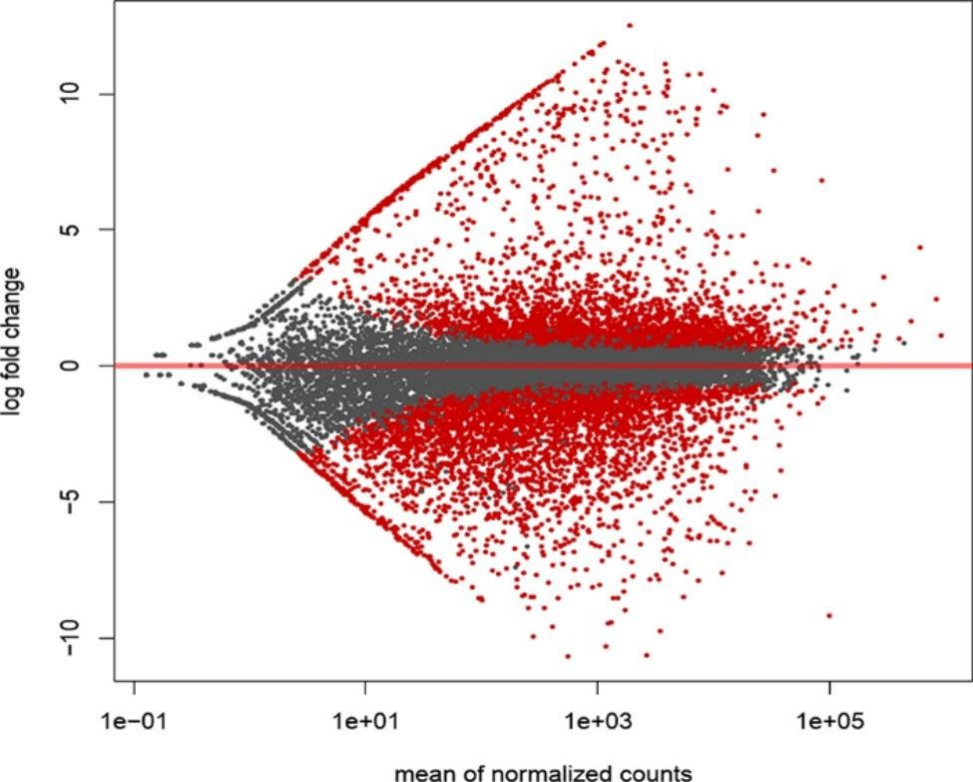
Differential expression of *C. rosea* strain ACM941 genes. Colors represent ACM941 genes significantly up- or down-regulated (red) and those that are not significantly regulated (black) in comparison to gene expression in strain 88–710 (set to zero). Volcano plot was obtained using the DESeq2 tool

Changes in gene expression underlying observed changes in the exometabolome have been shown to precede metabolite accumulation by up to several days [11]. Thus, toward investigation of the mechanisms underlying observed metabolic and functional differences at day-14, a comparison of ACM941 versus 88–710 strain gene expression profiles at day-11 was conducted. A total of 5,301 differentially regulated ACM941 genes (p < 0.05) were detected, of which 3,064 were upregulated (fold change (FC) ≥ 2.0) and 2,090 were downregulated (FC ≤ -2.0) (Fig. 4) compared to 88–710. The RT-qPCR results of selected genes correlated very well with the RNAseq, confirming reliability (Additional file 1: Fig. S2). To reduce the complexity of downstream analyses of upregulated and downregulated genes, only genes with average normalized read counts (RPKM) of |≥ 0.5| and fold changes (FC) of |≥ 5.0| were considered further. This reduced the number of up- and downregulated ACM941 genes compared to 88–710, to 460 and 301, respectively.

A number of SM biosynthesis and membrane transport related genes were among the most highly upregulated genes in ACM941 when compared to 88–710. For example, of the top 50 upregulated genes, 17 encode SM biosynthesis related proteins, 1 encodes a major facilitator superfamily homolog and 18 of them encode hypothetical proteins (Additional file 2: Table S1). The upregulated gene library was initially screened for non-ribosomal peptide synthase (NRPS) and polyketide synthase (PKS) anchored gene clusters (Additional file 3: Table S2 see NRPS and PKS Tabs), based on the recognized role of such metabolites in biocontrol. Interestingly, every gene member of a *C. rosea* putative epidithiodiketopiperazine (ETP) gene cluster was found to satisfy the stringent selection criteria for upregulation (average RPKM cut off value of 0.5 and FC ≥ 5.0), where ETPs are fungal alkaloids with proven cellular toxicity [29–31]. In particular, the ETP cluster anchor gene encoding an NRPS homolog protein, scf_095.g187 is among the most significantly upregulated genes detected in this study (RPKM = 37, FC = 70.1 and FDR p-value = 0). Two more NRPS homologs, scf_077.g266 (RPKM = 2.1, FC = 136, FDR p-value = 0) and scf_029.g101 (RPKM = 0.6, FC = 52 and FDR p-value = 3.29E-03) were also identified based on their putative role in *C. rosea in vitro* antagonism. Scf-077.g266 encodes a putative fumisoquin gene cluster anchor gene NRPS [7], also with all genes comprising the cluster upregulated. Another putative NRPS encoding gene scf_070.g317 (RPKM = 1.3, FC = 57.3 and FDR p-value = 0) satisfied the selection criteria but was not surrounded by associated upregulated genes. In addition, an uncharacterized cluster anchored by the NRPS homolog gene, scf_001.g638 (RPKM = 1.1, FC = 1.1, FDR p-value = 0.8) was identified as an upregulated gene cluster because surrounding genes met our stringent selection criteria even though the NRPS homolog did not.

Six gene clusters anchored by PKS genes were also identified in the upregulated transcriptomic library based on the expression level of PKS homologs meeting the selection criteria (Additional file 3: Table S2 PKS Tab). Of these, four were surrounded by associated upregulated genes. Scf_059.g219 (homolog to the highly reducing polyketide synthase alnA) was upregulated 242-fold (RPKM = 0.8, FDR p-value = 6.65E-08), while scf_023.g116 (homolog to the highly reducing polyketide synthase alt5; RPKM = 2.0, FDR p-value = 0) and scf_064.g288 (homolog to Lovastatin diketide synthase mokB; RPKM = 0.7, FDR p-value = 7.79E-15), were upregulated 187 and 161-fold in ACM941, when compared to 88–710, respectively. Similarly, scf_091.g78, a putative PKS homolog protein encoding gene, also met selection criteria (RPKM = 1.52, FC = 13.8 and FDR p-value = 2.14E-12). On the other hand, scf_004.g153 (RPKM = 0.07, FC = 12.6 and FDR p-value = 4.26E-03) was identified as part of an upregulated gene cluster because although it did not, surrounding genes did meet the selection criteria. Finally, scf_042.g174 (FC = 145.4 and FDR p-value = 9.52E-03) satisfied our selection criteria but wasn’t surrounded by upregulated genes.

### Functional annotation of the putative ***C. rosea*** ETP gene cluster

There were 14 upregulated genes in the *C. rosea* putative ETP gene cluster with homology to genes in known ETP gene clusters including verticillin from *C. rogersoniana* [29], chaetocin from *Aspergillus fumigatus* [30] and leptosin C from *Preussia typharum* [31] (Table 1). The first upregulated gene, scf_095.g183 (FC = 105.4, FDR p-value = 0), showed homology to the cytochrome P450 monooxygenase verB (verticillin cluster), chaB (chaetocin cluster) and lepB (leptosin C cluster). Scf_095.g185, a hypothetical protein homolog, didn’t have homologous counterparts in the verticillin and chaetocin gene clusters but showed 58% similarity to a hypothetical protein in the leptosin C gene cluster. Scf_095.g184, on the other hand showed 67 and 53% similarity with verZ and Gene15 of verticillin and leptosin C gene cluster members, respectively. Scf_095.g186 showed homology to two verticillin gene cluster members: verG and verJ and their counterparts in the chaetocin (chaG and chaJ) and leptosin C (lepG and lepJ) gene clusters. It is likely that the alignments of verG/chaG/lepG and verJ/chaJ/lepJ with scf_095.g186 don’t overlap, because scf_095.g186 is an assembly artifact of two genes predicted as one. In particular, verG, chaG and lepG aligned with the first 700 bp of scf_095.g186 while verJ, chaJ and lepJ were aligned with sequences after the 700th bp, respectively. Scf_095.g187, a 2374 aa long NRPS encoding gene, shows strong homology to verP, chaP and lepP with 75, 65 and 65% similarities, respectively. Further, nrps specific structural analysis on scf_095.g187 using antiSMASH fungal version [32] showed that scf_095.g187 contains both N and C-terminal peptidyl carrier proteins (PCPs), with dual-condensation (dual condensation/epimerisation domain) and adenylation (AMP-binding) domains in between in this respective order. This is consistent with the lepP gene from the leptosin C gene cluster which contains the N-terminal PCP, a condensation domain linking a L-amino acid to a peptide ending with a D-amino acid (condensation-DCL) and an AMP-binding domain in this respective order, but lacking the C-terminal PCP domain. The chaetocin gene cluster nrps homolog chaP, on the other hand, contains both PCPs and the condensation-DCL, AMP-binding, another PCP-binding, condensation domain linking an L-amino acid to a peptide ending with an L-amino acid (condensation-LCL) and a PKS-PP binding domain in this respective order.

**Table 1:** * C. rosea* putative ETP gene cluster annotation based on homology with other known fungal ETPs.

*C. rosea* putative ETP cluster member gene ID	Homolog in *P. trypharum* leptosin C gene cluster	Similarity (%)	Homolog in *C. rogersoniana* verticillin gene cluster	Similarity (%)	Homolog in *Gliocladium spp* chaetocin gene cluster	Similarity (%)	Putative function	Fold change	FDR p-value
scf_095.g183	ptlepB	66	crverB	90	chaB	80	Cytochrome P450 monooxygenase	105.3831962	0
scf_095.g184	Gene15	53	crverZ	67	-	-	Transcription factor	26.99595784	0
scf_095.g185	Gene16	58	-	-	-	-	Hypothetical protein	6.901224128	2.46E-09
scf_095.g186*	ptlepJ	82	crverJ	88	chaJ	79	Dipeptidase	7.985410071	7.79E-15
scf_095.g186*	ptlepG	89	crverG	93	chaG	90	Glutathione S-transferase	7.985410071	7.79E-15
scf_095.g187	ptlepP	65	crverP	79	chaP	65	Nonribosomal peptide synthetase	70.87984034	0
scf_095.g188	ptlepC	70	crverC	81	chaC	69	Cytochrome P450 monooxygenase	89.35346975	0
scf_095.g189	ptlepI	70	crverI	76	-	-	Aminotransferase	89.31396971	0
scf_095.g190	ptlepN	71	crverN	86	chaN	63	N-methyltransferase	89.14872847	0
scf_095.g191	ptlepM	81	crverM	91	chaM	78	O-methyltransferase	98.18660262	0
scf_095.g192	Gene11	87	crverL	91	chaE	50	Cytochrome P450 monooxygenase	98.08712138	0
scf_095.g193	ptlepT	76	crverT	89	chaT	83	Thioredoxin reductase	101.0477764	0
scf_095.g194	ptlepA	77	crverA	88	chaA	73	ABC-type transmembrane transporter	14.83370549	4.27E-12
scf_095.g195							Unknown	8.215857431	2.57E-10

‘*’ scf_095.g186 seems to be an artifact of two genes covering ptlepJ (49% coverage, aligns after first 700 bp) and ptlepG (36% coverage, aligns with the first 700 bp out of 1845), or covering crverJ (50% coverage, aligns after first 700 bp) and crverG (37% coverage, aligns with the first 700 bp out of 1845), respectively. ‘-‘: represents missing genes.

The remaining genes, scf_095.g188 – g194 showed 76–91% similarity to verC, verI, verN, verM, verL, verT and verA, respectively. Similarly, these proteins showed 76–87% similarity with lepC, lepI, lepN, lepM, gene11, lepT and lepA of the leptosin C gene cluster. With the exception of the putative aminotransferase encoding gene scf_095.g189, which did not find a homologous counterpart in the chaetocin gene cluster, the remaining genes exhibited 50–83% similarity with chaC, chaN, chaM, chaE, chaT and chaA (Table 1). Overall, the *C. rosea* putative ETP gene cluster members maintained significantly high sequence similarity with their verticillin gene cluster counterparts compared to those from leptosin C and chaetocin (Table 1). Interestingly, although *Gliocladium* spp are genetically closer to *C. rosea* than *P. trypharum*, the *C. rosea* putative ETP cluster members maintained high sequence similarity to their counterparts in both chaetocin and leptosin C gene clusters. Finally, scf_095.g195 that encodes for a hypothetical protein is missing from the three gene clusters.

Interestingly, the *C. rosea* putative ETP gene cluster members showed significantly lower similarity to other ETP biosynthetic gene clusters. For example the NRPS encoding gene of the scf_095.g187 showed only 37% and 28% similarity respectively with the gliotoxin gliP gene (the NRPS of the *Leptosphaeria maculans* glitoxin gene cluster), and sirodesmin sirP gene (the NRPS of the *A. fumigatus* sirodesmin gene cluster), compared to 79%, 65% and 65% similarity to *C. rogersoniana* verticillin cluster gene crverP, and *Gliocladium spp* and *P. trypharum* leptosin C cluster gene ptverP, respectively.

### IntLIM analysis

Experimental design of metabolomics and transcriptomics studies plays a critical role in reducing the complexity of multi‘omics data integration, particularly when concurrent metabolites and RNA extraction from the same sample is not practical. As recommended by Cavill et al., [33], source-matched or split sampling designs are the best strategy compared to replicate-matched or repeated experiments to generate metabolomics and transcriptomics data for integration. However, protein translation and subsequent biosynthesis and secretion of exometabolites takes some amount of time following transcription, such that integrating exometabolomics and transcriptomics data generated using source-matched or split sampling designs may not capture the relationship between metabolites and transcripts. On this basis, integration of day-11 RNAseq data with day-14 exometabolites was attempted. However, with data derived from two different experimental sets, batch effect irregularities were detected, which couldn’t be corrected using statistical methods. Similar discrepancies have been reported before as well [33, 34]. Therefore, a split sampling strategy with day-11 RNAseq and day-11 exometabolomics data derived from the same flask was implemented to reduce complexities arising from batch effect variability. This approach was validated by an observed increase in metabolite accumulation over the full 21-day fermentation period, where the top 75 highly accumulated metabolites derived from the same biological replicate (same flask) tended to cluster together regardless of sampling time (Additional file 1: Fig. S5). This is consistent with expected exometabolite data essentially providing a snapshot of an aggregated accumulation over the fermentation period due to metabolite stability. Variation in the PCA plot for day-11 exometabolomics data further emphasized batch effect variability and thus the value of split sampling (Additional file 1: Fig. S6A).

Initial filtering of day-11 RNAseq expression profiling data (ACM941 compared to 88–710) using IntLIM default parameters excluded the lowest 10% expressing genes yielding 15,826 genes to proceed with (Additional file 1: Fig. S7A). There were no exometabolites filtered by IntLIM from day-11 samples using the default exclusion criteria (80% imputed values) as metabolites that did not meet these constraints were already removed by MetaboAnalystR software during normalization (Additional file 1: Fig. S7B). The first order principal component analysis (PC1 = 68.4%) showed good separation between *C. rosea* strain ACM941 and 88–710 transcriptomic data (Additional file 1: Fig. S6B). However, replicate 1 of ACM941 exometabolites was an outlier at PC1, 54.5% of the variation (Additional file 1: Fig. S6A), because the overall metabolite concentration of ACM941 replicate 1 was very low compared to all other ACM941 and 88–710 replicates. Despite this variation, at PC2 (27.61% of the observed variation), all ACM941 replicates did cluster separately from 88 to 710 replicates. Based on their genomic similarity level, ACM941 and 88–710 are expected to produce similar metabolites with more subtle differences in accumulation patterns dictating their phenotype. Therefore, we decided to continue with the analysis as the PC2 separation pattern was deemed relevant. IntLIM correlation analysis, with a p-value cut off set at 0.05 and correlation difference of 0.5, produced 1,589,504 gene-metabolite pairs (Additional file 1: Fig. S8A). With further filtering of the output using a p-value cut off of 0.01 and correlation difference of 0.05, the pairing was reduced to 2,679 gene-metabolite pairs (Additional file 1: Fig. S8B).

**Fig. 5:**
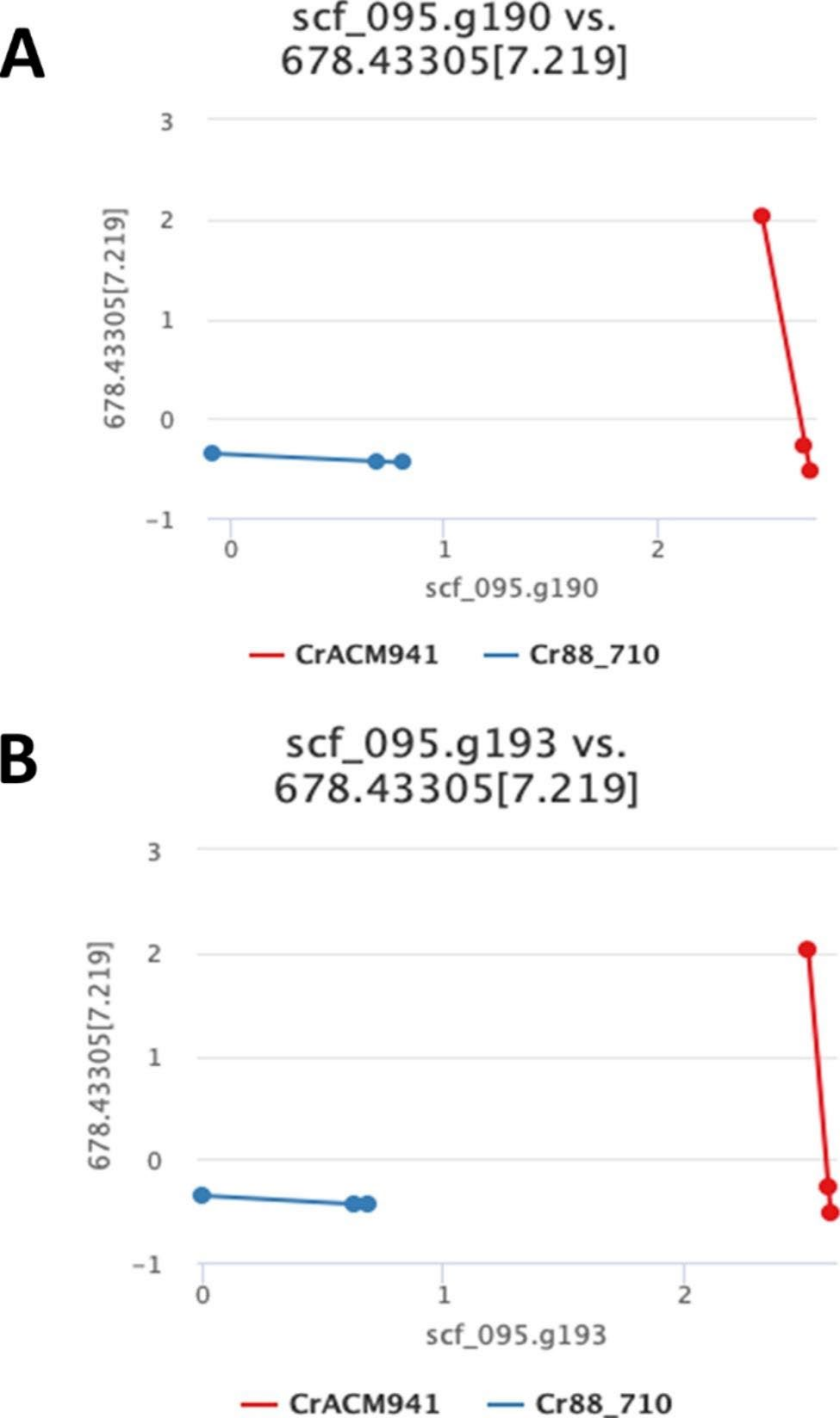
IntLim identified gene-metabolite pairs. Scatter plot visualization of selected gene-metabolite pairs, colour codes red (ACM941) and blue (88–710). **(A)** NRPS anchored cluster gene scf_095.g190 vs. metabolite MW 678.43305[7.219] (ACM941 corr. = -1; 88–710 corr. = -0.5; p-value = 0.0001374360) and **(B)** NRPS anchored cluster gene scf_095.g193 vs. metabolite MW 678.43305[7.219] (ACM941 corr. = -1; 88–710 corr. = -0.5; p-value = 0.0001055869)

On the assumption that *C. rosea* mycoparasitism against *F. graminearum* is likely determined by SMs, gene-metabolite pairings linked with SM encoding gene clusters were targeted for further analysis. We screened the gene-metabolite pairs table for NRPS anchored gene clusters (described above) that showed significant linear correlation with selected metabolites, paying special attention to metabolites possibly linked to the upregulated putative ETP gene cluster identified by IntLIM. In this regard, two genes from the cluster anchored by scf_095.g187 (the putative NRPS encoding gene in the *C. rosea* ETP cluster), showed a significant correlation. In this instance, the methyltransferase homolog scf_095.g190 (Fig. 5A) and the thioredoxin reductase homolog scf_095.g193 (Fig. 5B) showed strong correlation with the metabolite *m/z*_*obs*_ 679.43774 ([M + H]^+^) in the extraction ion chromatogram (EIC; centered within a 5 ppm window) that eluted at 7.23 min (Additional file 1: Fig. S9). Interestingly, another metabolite with *m/z*_*obs*_ 621.43481 ([M + H]^+^), was found to co-elute with the *m/z*_*obs*_ 679.43774 ([M + H]^+^) at 7.23 min (Additional file 1: Fig. S10). Both of these metabolites showed a doubling trend every 7 days. The nature of the structural relationship between these two compounds remains to be determined.

### Genome level similarity analysis

**Fig. 6:**
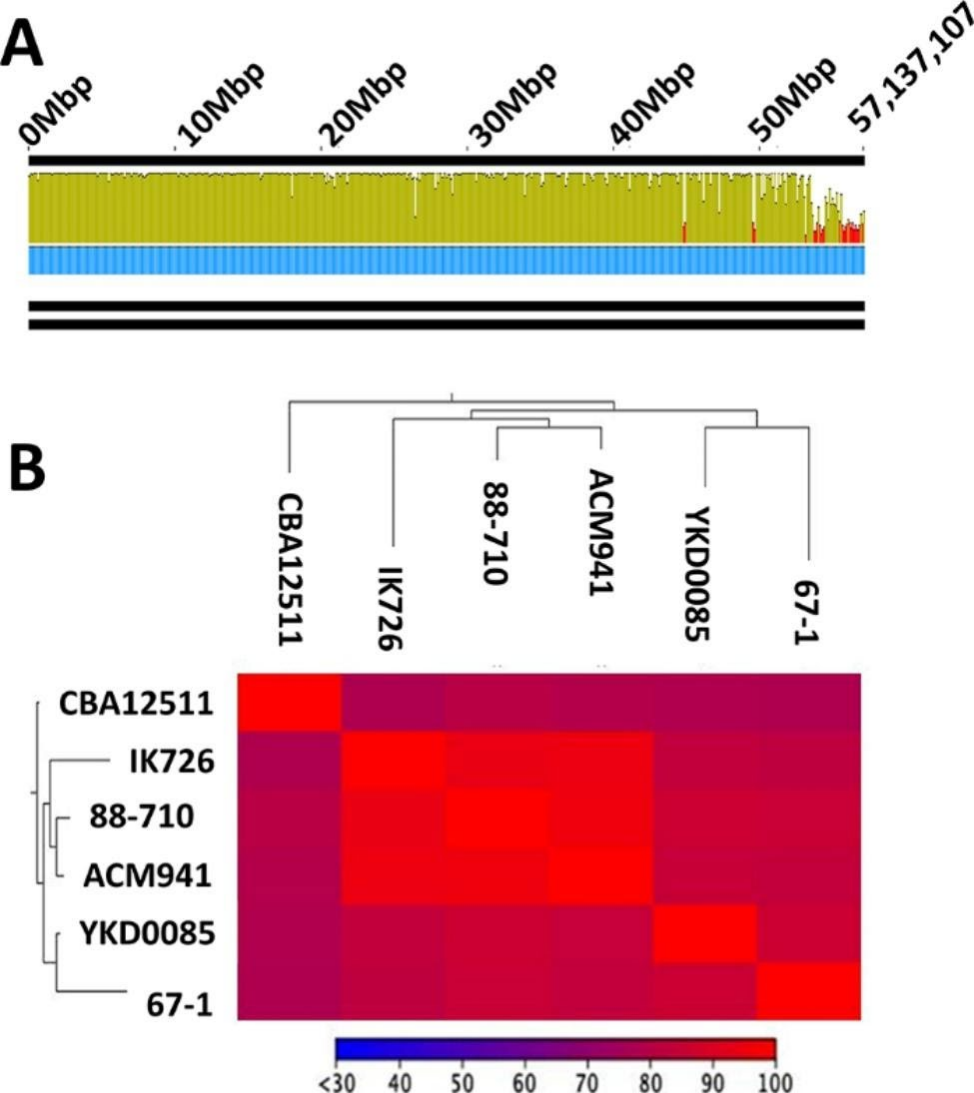
Genetic relationship among *C. rosea* strains ACM941. **A) ***C. rosea* strain ACM941 whole genome (bottom panel) aligned against that of 88–710 genome (top panel) using progressiveMauve. Colours denote mean pair wise identity where green is 100%, green-brown is at least 30% and under 100% identity and red represents less than 30% identity. The blue line shows coverage. **B)** Heatmap of multiple alignment among whole genome sequences of all sequenced strains and

Differences between *C. Rosea* strain ACM941 and 88–710 genomes were restricted to a few selected contigs (Fig. 6A). Furthermore, genome level phylogenetic analysis also showed that the three *C. rosea* strains (ACM941, 88–710 and IK726) were rooted closely followed by *C. rhizophaga* strain YKD0085 and *C. chloroleuca* strain 67 − 1, while *C. solani* strain CBS 2511 was rooted separately (Fig. 6B).

## Discussion

The functional and geographic overlap of *C. rosea* strain ACM941 and 88–710 is supported by their high genetic similarity level compared to all other sequenced *C. rosea* strains (Fig. 6B – genome phylogeny). The pairwise genome comparison between *C. rosea* strain ACM941 and 88–710 also revealed that their differences were largely restricted to few scaffolds (Fig. 6A – genome alignment). This was in agreement with previous reports where the genetic similarity between ACM941 and 88–710 was described using different molecular methods [4, 5]. In particular, previous reports showed that the ACM941 genome harbours genes encoding one unique NRPS, one unique hybrid and two unique PKS genes which were missing in 88–710, while the 88–710 genome encoded two unique PKS homologs [5]. In this study, comparative ‘omics techniques were applied in an attempt to discern any metabolic differences between the two strains that derive their in vitro antagonism potential against *F. graminearum*. Not surprisingly, their genomic variations are consistent with the observed deviation in terms of both unique and total number of metabolites detected between ACM941 and 88–710 on days 14 and 21. For example, at day-14, 28 and 6 unique metabolites were detected in ACM941 and 88–710, respectively, although, these were reduced to 10 and 4 respectively, by day-21 (Additional file 4: Table S3). Interestingly, in vitro antagonistic differences between the 2 strains were only observed in day-14 extracts (Fig. 1). This implies that the differential *F. graminearum* growth inhibition observed with the day-14 exometabolites, is likely mediated by exometabolite(s) showing delayed accumulation levels in 88–710, compared to ACM941, rather than unique metabolites (Fig. 2). On this basis, focus was shifted from comparative genomics to comparative global expression profiling of ACM941 and 88–710. In particular, significantly upregulated fungal NRPS and PKS anchored SM gene clusters were considered, leading to the identification of five NRPS and six PKS anchored gene clusters (Additional file 3: Table S2). However, it is wroth to note that although ACM941 and 88–710 were cultivated in SM inducing media, *C. rosea* and *F. graminearum* contact induced metabolic differences deriving their mycoparasitism are beyond the scope of this study.

Correlation and logistic regression-based transcriptomic and metabolomic data integration approaches generally capture co-regulation patterns to predict molecular interactions or phenotypic effects [15–25]. In contrast, IntLIM assumes co-regulation of functionally related genes and metabolites [35, 36] to predict novel phenotype specific gene-metabolite interactomes irrespective of well characterized genomic and metabolomic information [27]. We opted to use IntLIM to identify in vitro antagonistism-specific gene-metabolite relationships that can potentially describe the strong in vitro antagonistism of ACM941 against *F. graminearum* because *C. rosea* lacks a well curated genomic and metabolomic database. One disadvantage of such numerical-based data integration approaches is associating multiple metabolites with multiple transcripts. In addition, linear metabolite-gene associations don’t consider the fact that metabolite abundance is dependent on several structural enzymes along a pathway as well as many associated regulatory proteins. Therefore, we attempted to minimize this complexity by focusing our data analysis and interpretation steps on members of upregulated gene clusters showing strong association with upregulated metabolites. Of the five NRPS anchored gene clusters satisfying our stringent upregulation criteria (Additional file 3: Table S2), one NRPS gene cluster was found to have significant correlation with different metabolites. In particular, two genes belonging to the putative *C. rosea* ETPs gene cluster garnered our attention (Fig. 5).

Transcript abundance and metabolite synthesis do not necessarily have linear relationships. In particular, it is difficult to conclusively associate untargeted metabolomics data, which are prone to mis-identification of metabolites, with transcripts. The nature of untargeted metabolomics data further complicates the interpretation of gene-metabolite linkages because it is difficult to differentiate between intermediary and final products of a gene cluster as well as adduct metabolites. For example, the hypothetical molecular weight of the metabolite (678.43305) associated with the methyltransferase and thioredoxin reductase homologs scf_095.g190 and scf_095.g193, respectively, from the putative *C. rosea* ETP cluster is lower than that of known ETPs: verticillin A (MW 696.8), chaetocin (MW 696.8) and leptosin C (MW 740.9). In addition, the transcript abundance of scf_095.g190 and scf_095.g193 and accumulation of the metabolite with MW 678.43305 showed strong negative correlation (Fig. 5). We identified this relationship as one of the candidates for further investigation based on two hypotheses: firstly gene homology was given emphasis over the predicted relationship type and secondly the metabolite could be an intermediary product produced and consumed along the pathway. However the possibility that it is not associated with this cluster at all cannot be excluded. Indeed, most candidate genes and clusters identified in this study require experimental validation of the type of metabolite they produce, as well as their role in mediating ACM941 in vitro antagonistic properties against *F. graminearum*. Similar untargeted metabolomics data has been successfully used to model the transcript-metabolite associations in maize using the WGCNA software [18].

The *C. rosea* ETP gene cluster (Table 1) was identified as a primary target based on differential expression and data integration results. The putative gene cluster showed highest sequence similarity with the verticillin gene cluster from *C. rogersonian* [29], followed by the leptosin C cluster from *P. typharum* [31] and then the chaetocin gene cluster from *Gliocladium* spp [30]. It is worth noting that verticillin, chaetocin and leptosin C, are all structurally dimeric ETPs (Table 1). In contrast, gene cluster sequence similarity was much lower to the two known gene clusters that make the structurally monomeric ETPs, gliotoxin and sirodesmin. Thus, the putative *C. rosea* ETP gene cluster is more likely producing a dimeric ETP. Speculatively, the *C. rosea* putative ETP gene cluster product is more likely to produce leptosin C because both it contains an additional homolog gene which is not present in the verticillin gene cluster. In addition, structural analysis of the putative NRPS scf.095.g187 and its respective homologs showed that scf.095.g187 functional module types and arrangements show better similarity to lepP than chaP. antiSMASH predicted that scf.095.g187 contains PCP, dual condensation/epimerisation domain, AMP-binding and PCP domains in this order, while the lepP gene contains PCP, condensation-DCL and AMP-binding domains in this order, respectively. This is in contrast to chaP, which maintains the 3 domains identified in lepP, but also contains additional domains including PCP-binding, condensation-LCL and PKS-PP. antiSMASH failed to detect any domains for verP. Nonetheless, the *C. rosea* ETP is likely a variation of leptosin C, because it contains an additional entirely novel hypothetical protein.

**Fig. 7:**
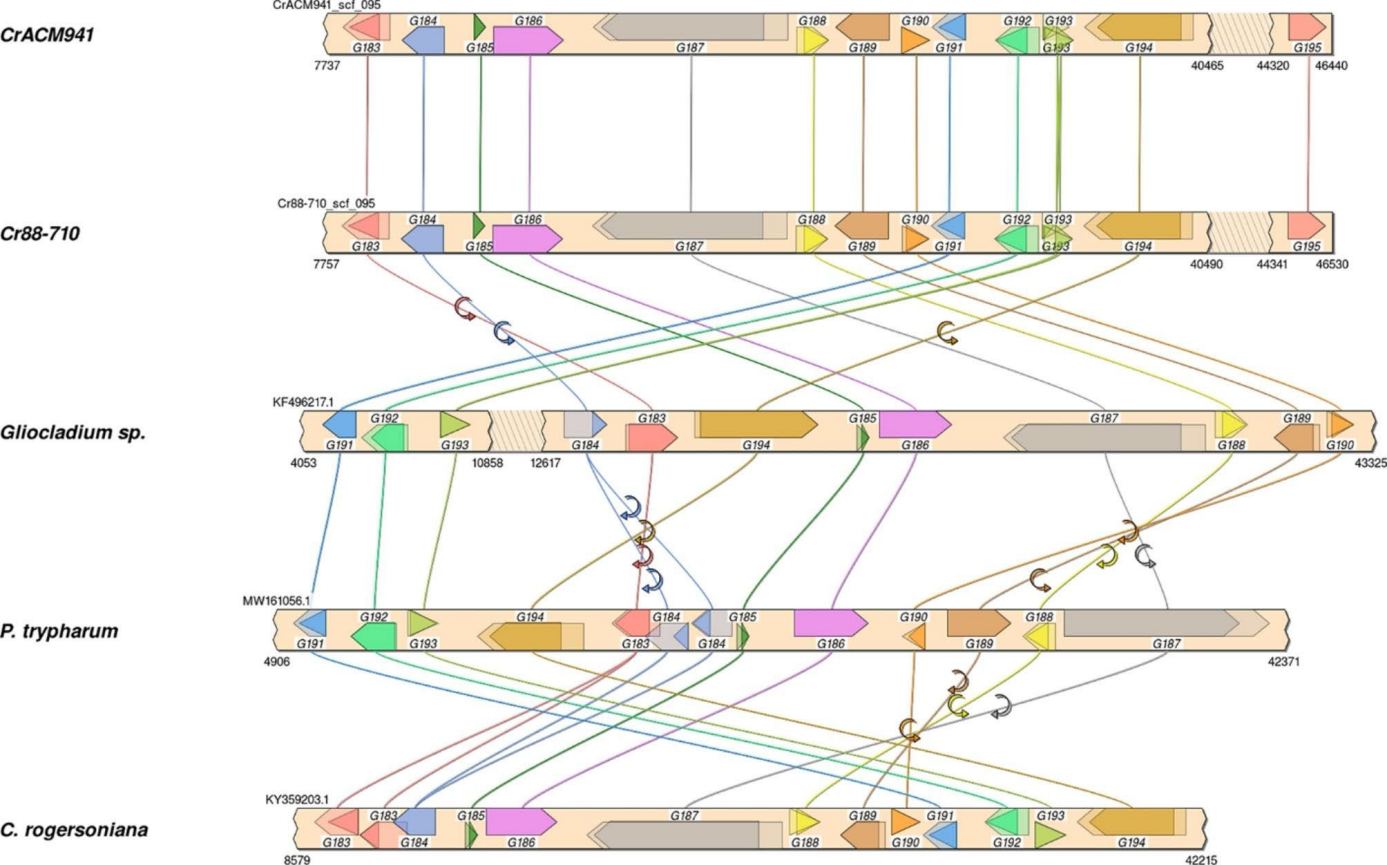
Graphic representation of putative and proven ETP-type gene clusters and their microsynteny relationship. *CrACM941* (*C. rosea* strain ACM941 putative ETP, this study), *Cr88-710* (*C. rosea* strain 88–710 putative ETP, this study), *Gliocladium* spp. (chaetocin gene cluster from *C. virescens* strain ATCC 26,417 [40]), *P. trypharum* (leptosin C gene cluster from *P. typharum* [41]), and *C. rogersoniana* (verticillin gene cluster from *C. rogersoniana* [39])

Finally, we mapped the *C. rosea* putative ETP gene cluster against genomic regions of verticillin [29], chaetocin [30] and leptosin C [31] gene clusters to evaluate their micro-syntenic relationship using SimpleSynteny [37]. The results showed that except for a few missing regions for select genes that did not have homologs in the other respective gene clusters (Table 1), the differences were largely dominated by genomic region rearrangements. Consistent with their sequence similarity level (Table 1), the verticillin gene cluster from *C. rogersoniana* [29] maintained a similar genetic arrangement with *C. rosea* putative ETP, while chaetocin [30] and leptosin C [31] gene clusters showed similar genomic arrangement between each other (Fig. 7).

ETPs are endowed with a myriad of biological activities including antifungal, anticancer, antibacterial and antiviral properties, and subject to intensive chemical synthesis research endeavors [38, 39]. The toxicity of ETPs is attributed to the redox properties of their disulfide bond, inhibition of thioredoxin reductase and histone methyltransferase, ejection of zinc ions from transcription factors as well as other mechanisms [30, 40, 41]. For example, leptosin C was shown to possess strong anti-cryptococcal activity against yeasts [31], by inhibiting DNA topoisomerases I and/or II and inducing apoptosis by inactivation of Akt/protein kinase B [42]. As well, gliotoxin has been shown to regulate the in vitro antagonistic property of the biocontrol agent *T. virens* against fungal pathogens including *Pythium ultimum* and *Rhizoctonia solani* [43, 44]. *C. rosea* also contains a putative gliotoxin biosynthetic gene cluster whose expression was not modulated (data not shown). Therefore, it is not a surprise that a homolog of such gene clusters is among the most upregulated gene clusters potentially modulating the in vitro antagonistism of *C. rosea* strain ACM941 compared to strain 88–710.

## Conclusions

We generated transcriptomic and metabolomic data of *C. rosea* strain ACM941 and 88–710 to elucidate the genetic and metabolomic underpinnings of their in vitro antagonistism ability. Specialized metabolism and membrane transport related genes were among the significantly upregulated genes in ACM941 relative to 88–710. In addition, high molecular weight SMs were differentially secreted and the accumulation pattern of some metabolites matches the growth inhibition differences displayed by the exometabolites of the two strains. In an attempt to identify statistically relevant relationships between upregulated genes and differentially secreted metabolites, transcript and metabolomic abundance data were associated using IntLIM. Of the several testable candidate associations, a putative *C. rosea* ETP gene cluster was identified as a prime candidate based on both co-regulation analysis and transcriptomic-metabolomic data association. Despite the need for additional validation by gene deletion or overexpression, our result suggest that a data integration approach may be used to identify potential biomarkers mediating the intricate functional divergence between *C. rosea* strains ACM941 and 88–710.

## Materials and methods

### Comparative genomics

*C. rosea* strain ACM941 and 88–710 whole genomes [5] were aligned using progressiveMauve software with default parameters and HOXD default scoring matrixes [45]. Aligned genomes were then visualized with Geneious Prime® 2021.1.1. Alternatively, the alignment coordinates of progressiveMauve output were extracted using Circos: Alignments to Links software in Galaxy (Galaxy Version 0.69.8 + galaxy7, [46]) converted into circular alignment figures and visualized using Circa (Version 1.2.2 (1.2.2)). SimpleSynteny version 1.5 was used to produce the graphical representation of syntenic relationships among gene clusters with the following settings: gapped alignment with BLAST E-value = 0.001 and Minimum query coverage cutoff = 30%.

### ***F. graminearum*** growth inhibition by ***C. rosea*** ACM941 and 88–710 exometabolites

*C. rosea* strains ACM941 and 88–710 were maintained as described previously [7]. Four agar plugs of actively growing *C. rosea* ACM941 and 88–710 mycelia (harvested using the wide end of a 1 mL sterile pipette tip for consistency) were inoculated into 100 mL Czapek-Dox broth (HiMedia, USA), in 500 mL baffled flasks and incubated at 25 °C with shaking at 180 rpm. After 7, 14 and 21 days of incubation, fungal mass was pelleted by centrifugation at 4,000 × g for 10 min at 4 °C and secreted metabolites (exometabolites) were partially purified from the supernatant using amberlite XAD-2 resin (Sigma Aldrich, Canada). Resin was added to the supernatant (1 g per 100 mL culture broth) and incubated on a rotating platform at 4 °C overnight. The mixture was loaded into a column where the flow-through was discarded, the resin was washed with three initial culture volumes of ice-cold water and the remaining metabolites eluted with methanol in 1 mL fractions. Extracts were further concentrated by evaporation of approximately 75–80% of the methanol using a CentriVap vacuum concentrator. The concentrated eluate (100 µL) was applied to a filter disc for growth inhibition assays. After the indicated times of 7, 14 or 21 days the area forming the growth inhibition zone quantified using ImageJ and plotted as a percent of the area comprising 1/3 of the total area of the plate.

### ***C. rosea*** ACM941 and 88–710 exometabolome profiling

For each time point (7, 14 and 21 day), three types of samples were prepared, including samples of the spent *C. rosea* fermentation medium, unused (fresh) fermentative medium as a background control, and procedural blanks consisting of methanol only. At each time point, three biological replicates of *C. rosea* cultures were set aside for LC-MS analysis. Five individual samples of each sample type were prepared to provide five independent technical replicates on the LC-MS. All analyses were performed on a Vanquish UHPLC in tandem with an Orbitrap Fusion Lumos mass spectrometer. High resolution mass spectral data were recorded in positive mode at a resolution of 120 000, a maximum linear fill trap time of 100 ms and scan ranges of *m/z* = 250–2000 amu respectively. Samples were diluted 100-fold, prior to injection of a 2 µL sample on the LC-MS. Tuning parameters for the heated electrospray ionization (H-ESI) source was as follows: RF lens 55% V, capillary temperature 300 °C, spray voltage 3.5 kV, sheath gas flow 45 units, auxiliary gas flow 20 units and sweep gas flow 2 unit. Elution by UHPLC proceeded at 0.3 mL/min on an ACE-C18-PFP column (C18, 50 × 2.1 mm, 2 μm) heated to 40 °C, using a mobile phase consisting of acetonitrile:water modified with 5 mM ammonium acetate and 0.1% formic acid, and the following gradient: 0–0.5 min (30% acetonitrile), 0.5–8 min (30 to 95% acetonitrile), 8–11 min (95% acetonitrile), 11.5–12 min (95 to 30% acetonitrile) and 12–15 min (30% acetonitrile).

Differential analysis of *C. rosea* culture samples compared to the unused medium controls were performed with Compound Discover 2.0 (ThermoFisher, CA). The .RAW files were obtained in full MS mode and were processed directly by the software using a built-in workflow for untargeted metabolomics. Compounds were detected by retention time, a minimum peak intensity of 100 000 and a mass tolerance of 5 ppm. Peak areas from all technical replicates were normalized to account for instrument variability. The peak intensities of the detected compounds were compared between the *C. rosea* culture samples and the plain medium control samples. Compounds displaying a Log2-fold increase of > 9 in the *C. rosea* samples compared to the plain medium control were considered relevant.

### ***C. rosea*** ACM941 and 88–710 transcriptome profiling

Four plugs of actively growing *C. rosea* ACM941 and 88–710 mycelia were inoculated into 500 mL baffled flasks containing 100 mL Czapek-Dox broth (HiMedia, USA) and incubated at 25 °C with shaking at 180 rpm for 11 days. Fungal mass growing on the side of the flask just above the media was isolated for RNA extraction and flash frozen with liquid nitrogen. Genomic DNA-free high-quality total RNA was extracted from frozen mycelia using a combination of TRIzol® reagent (Invitrogen™) and InviTrap® Spin Universal RNA mini Kit (Stratec molecular, Germany). Briefly, freeze-dried fungal mass was ground to a fine powder and homogenized in 1 mL TRIzol solution followed by genomic DNA removal and total RNA isolation using InviTrap spin columns following the manufacturer’s protocol. Immediately following, the RNA concentration and purity was determined using a Nanodrop spectrophotometer ND-1000 (Thermo Scientific), and its integrity was confirmed by agarose gel electrophoresis. Total RNA (4 µg/sample) was shipped to the National Research Council of Canada, DNA Sequencing Technologies Facility (Saskatoon, Canada) where an additional quality check was performed using a BioAnalyzer followed by short cDNA fragment synthesis using TruSeq Stranded RNALT kit (Illumina, USA), and finally sequenced on an Illumina HiSeq 2500 platform according to the manufacturer’s guidelines (Illumina, USA). Full RNAseq data is available from the NCBI (Bioproject ID PRJNA916464). Concurrently, submerged mycelium was separated from the supernatant by centrifugation at 4,000 x g for 10 min at 4 °C and used for day-11 exometabolite extraction and profiling as described above.

### Differential gene expression analysis

Low quality short reads, adapter and other Illumina-specific sequences were filtered using Trimmomatic software v0.36.4 (http://www.usadellab.org/cms/index.php?page=trimmomatic) with the following modifications to the default settings: leading low quality cutoff was 17; sliding window was 5 bp with a minimum average quality score of 20, and read length cutoff was 60 bp instead of 36 bp [47]. Trimmed *C. rosea* strain ACM941 and 88–710 RNA-Seq data were aligned to the *C. rosea* strain ACM941 genome annotated with genes and transcripts [5] and used to calculate differential gene expression using the CLC Genomics workbench version 20.0 (Qiagen Corp.). Read alignment was performed using high stringency criteria: similarity fraction = 0.95 and length fraction = 0.8, mismatch cost = 2, deletion and insertion costs = 3 and maximum number of hits per read = 10. Gene expression levels were estimated as transcripts per million (TPM) [48] which was calculated as: *TPM = (RPKM x 10*^*6*^*) / Σ RPKM*, where the sum is over the *RPKM* values of all genes/transcripts. Reads per kilobase of exon model per million mapped reads (RPKM) [49] was calculated following the formula: RPKM = total exon reads / [mapped reads (millions) × exon length (KB)]. Differential gene expression or the ‘exact test’ [50] was implemented to compare ACM941 versus 88–710 *C. rosea* strains. Transcripts were considered as significantly expressed when fold change was ≥ 2.0 and the false discovery rate (FDR) was set at *p* < 0.05. Alternatively, trimmed *C. rosea* strain ACM941 and 88–710 RNAseq reads were mapped to ACM941 and 88–710 genome [5] using the read mapper software HISAT2 [51]. Differential expression of genes was calculated using HISAT2 alignment as an input in the following pipeline: gene and transcript counts in each sample were counted using Stringtie [52, 53] followed by differential gene/transcript abundance estimation using DESeq2 [54].

The RNAseq gene expression results were confirmed by qPCR, with prime IDT primer quest software (https://www.idtdna.com/Primerquest/Home/Index) targeting 190–200 base-pair (bp) fragments. cDNA was synthesized from total RNA stored at − 80 °C (remaining from sequenced samples) using the 5X All-In-One RT MasterMix Kit (ABM Inc., Canada). The relative transcript abundance of the selected genes were assessed by IQ™5 multicolour real-time polymerase chain reaction (PCR) detection system (Bio-Rad, USA) using the EvaGreen Express 2X qPCR MasterMix (ABM Inc., Canada) along with approximately 150 ng of cDNA template and 500 nM of each of the primers in a 20 µL reaction volume. The following program was used for real time quantitative PCR (RT-qPCR): 95 °C for 1 min followed by 40 cycles of 5 s at 95 °C and 15 s at 59 °C. Normalized relative expression values (ΔΔC_T_) of the selected candidates were calculated using the formula 2^−ΔΔ*CT*^ [55], with *EF1α* and *β-actin* as reference genes [56]. Serial dilutions of cDNA samples (10^0^ – 10^− 5^) were used to develop standard curves and confirm primer efficiency.

### IntLIM analysis

Metabolomics and gene expression data were pre-processed for IntLIM analysis based on the “NCItestinput” data format requirements [27]. *C. rosea* strain ACM941 and 88–710 compounds (n = 5 technical and n = 3 biological replicates) detected by Compound Discover 2.0 (ThermoFisher) were normalized using MetaboAnalystR [19] with the following criteria: replace sample missing values by column(sample) minimum value, missing features were replaced by the minimum value in the replicate, normalized by median, log transformed, and auto-scaled. Scaling was mean-centered and divided by the standard deviation of each variable. Metabolites with more than 50% imputed values were filtered resulting in input data containing 107 metabolites (identified by compound mass and retention time). Normalized metabolomic data and the list of differentially expressed data containing 17,585 transcripts were then organized using the NCI-60 data format and loaded into the IntLIM software [27]. An arbitrary cutoff value (10%) was used to filter the lowest expressing genes resulting in a total of 15,826 genes. Finally, a total of 107 metabolites and 15,826 genes were integrated using the linear model data integration pipeline of IntLIM R package. *C. rosea* strain ACM941 was compared with strain 88–710 to identify putative in vitro antagonistism related metabolite-gene pairs [27]. R version 4.0.3 (2020-10-10) was used for the analysis.

## Electronic supplementary material

Below is the link to the electronic supplementary material.

Additional file 1: Supplementary Figures S1-S10.

Additional file 2: table S1. Top 50 upregulated genes in *C. rosea* strain ACM941 relative to 88–710 and their putative function. Genes predicted to play a role in SM and transport are shaded in green.

Additional file 3: table S2. Selected non-ribosomal peptide synthase (NRPS Tab) and polyketide synthase anchored (PKS Tab) gene clusters upregulated in *C. rosea* strain ACM941 relative to 88–710. Anchor genes are shaded in green for NRPS and yellow for PKS.

Additional file 4: table S3. Differentially accumulated metabolites by *C. rosea* strain ACM941 and 88–710 after 14- and 21 days fermentation relative to day-7 samples. Metabolites detected only in ACM941 were shaded with green or yellow, while those detected only in 88–710 were shaded with blue.

Additional file 5: table S4. List of qPCR primers used in this study.

## Data Availability

The datasets generated and/or analysed during the current study are available in the NCBI BioProjects repository ID PRJNA916464 at the following persistent link: PRJNA916464 and the Sequence Read Archive at the following SRA persistent links: SRR22905843SRR22905844SRR22905845SRR22905846SRR22905847SRR22905848.

## List of Abbreviations

C. rosea: *Clonostachys rosea*
ETP: Epidithiodiketopiperazine
IntLIM: Integration through Linear Modeling
