# Supplementary material for: Clonostachys rosea ‘omics profiling: identification of putative metabolite-gene associations mediating its in vitro antagonism against Fusarium graminearum
Source: BMC Genomics. 2023 Jun 26;24:352. doi: 10.1186/s12864-023-09463-6 (PMC10291803; doi:10.1186/s12864-023-09463-6)
Supplement: Supplementary file 1 — Additional file 1: Supplementary Figures S1-S10. [file 12864_2023_9463_MOESM1_ESM.pdf]

Clonostachys rosea ‘omics profiling: identification of putative metabolite-gene associations mediating mycoparasitic properties against *Fusarium graminearum*

Adilah Bahadoor[1], Kelly A. Robinson[2], Michele C. Loewen[2*], Zerihun A. Demissie[2*]

[1]Metrology Research Center, National Research Council Canada, 1200 Montreal Rd, Ottawa, ON K1A 0R6 Canada

[2]Aquatic and Crop Resource Development, National Research Council of Canada, Ottawa, ON, Canada

Supplementary Figures S1 – S10

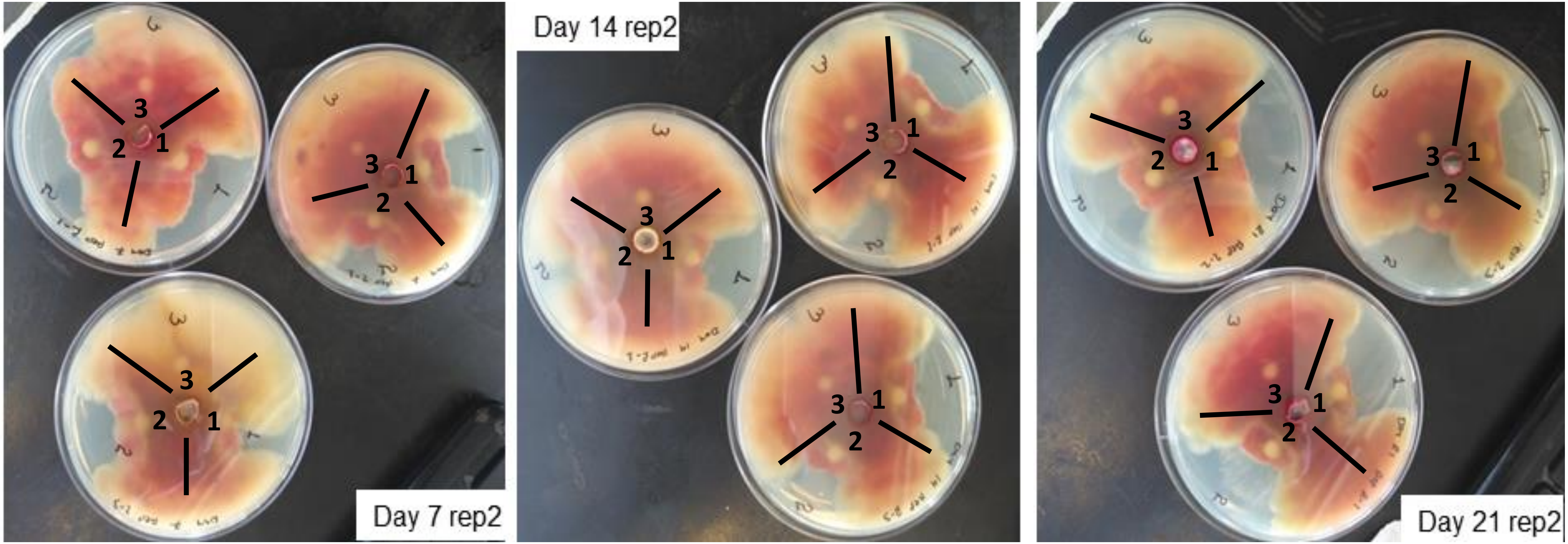

Fig. S1. Representative images that were used to quantify the growth inhibitory activity of exometabolites derived from C. rosea spent media after 7, 14 and 21 days of culturing. Growth inhibition of *F. graminearum* strain GZ3639 by exometabolites of *C. rosea* strain ACM941 (1) and 88-710 (2) fermented in Czapek Dox media as indicated. These were compared to methanol-only negative control (3). Zones of inhibition were quantified using ImageJ. Sample plates for n = 3.

(A)

(B)

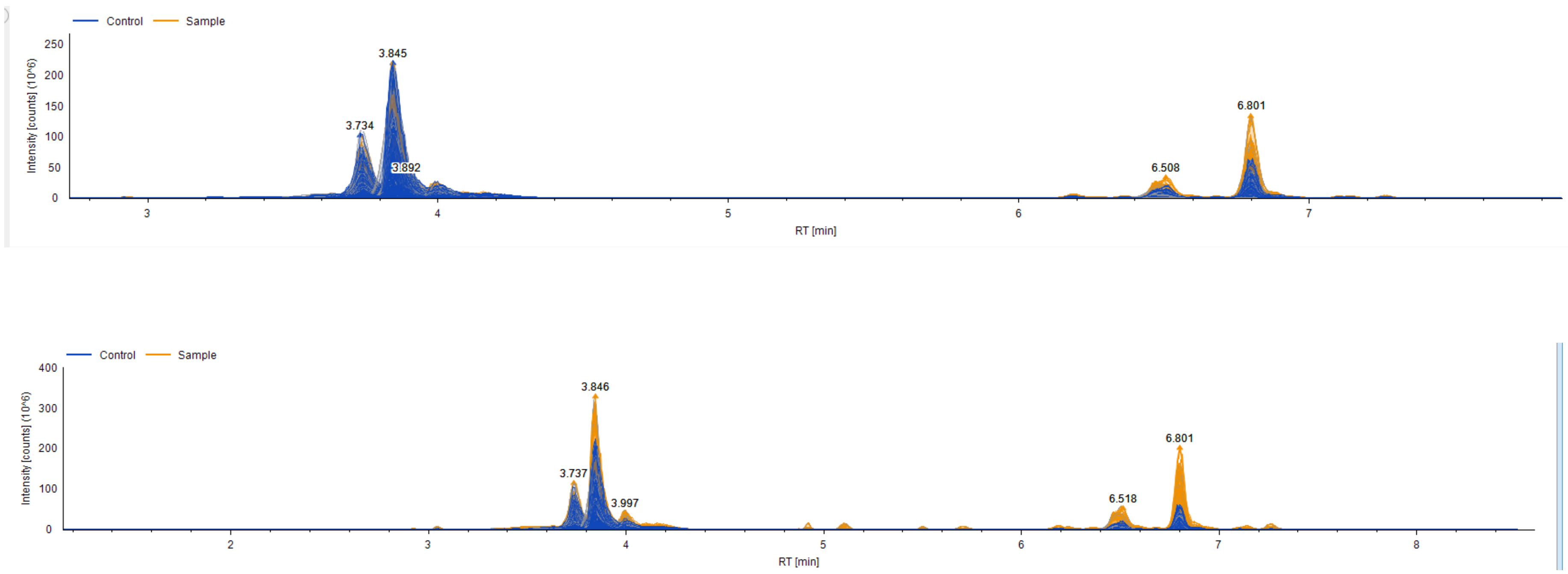

Fig. S2. The differential exometabolite profile of strain ACM-941 over 21 days. (**A**) Day 14 strain ACM-941 extracts (sample-orange) vs D7 extracts (control-blue) shows increase metabolite production for compounds eluting at 6-7.5 min, as opposed to compounds eluting earlier in the range 3.5-4.5 min. (**B**) Day strain 21 ACM-941 extracts (sample-orange) vs D7 extracts (control-blue) shows additional increase metabolite production for compounds at 6-7.5 min range.

(A)

(B)

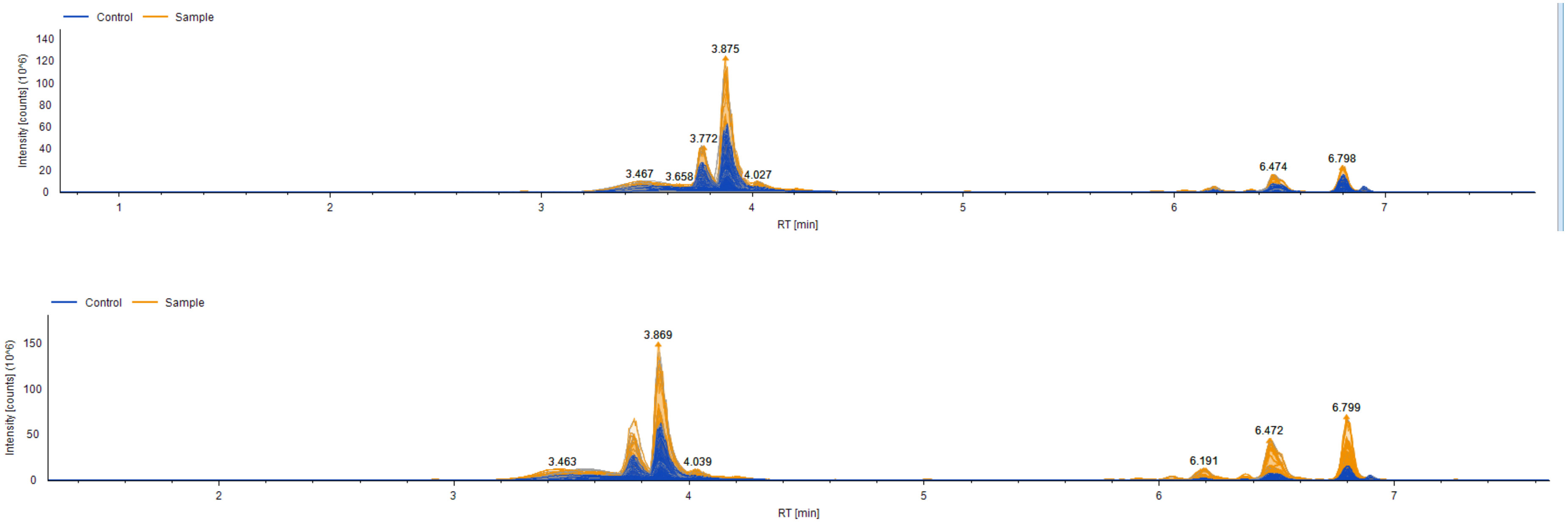

Fig. S3. The differential exometabolite profile of strain 88-710 over 21 days. (**A**) Day 14 strain 88-710 extracts (sample-orange) vs D7 extracts (control-blue) shows muted increase in the of production for compounds eluting at 6-7.5 min range. (**B**) Day 21 strain 88-710 extracts (sample-orange) vs D7 extracts (control-blue) shows clear increase in metabolite production for compounds at 6-7.5 min range.

Fig. S4. Validation of RNAseq results by qPCR. Results arising for a number of selected unchanged, upregulated and downregulated transcripts are shown. Gene ID description scf: 271.g103: , 291.g163: , c_3.g97: , c_291.g117: , 298.g180: , 027.g180: , 050.g111: , 014.g324: , 003.g301: , 289.g159: , 092.g104: , 092.g101: , 068.g206: , 049.g267: , 015.g172: , 027.g161: , 032.g327: , 001.g913: and 097.g246: . RNAseq outcomes are represented by black circles, qPCR outcomes are represented by circles with a central cross.

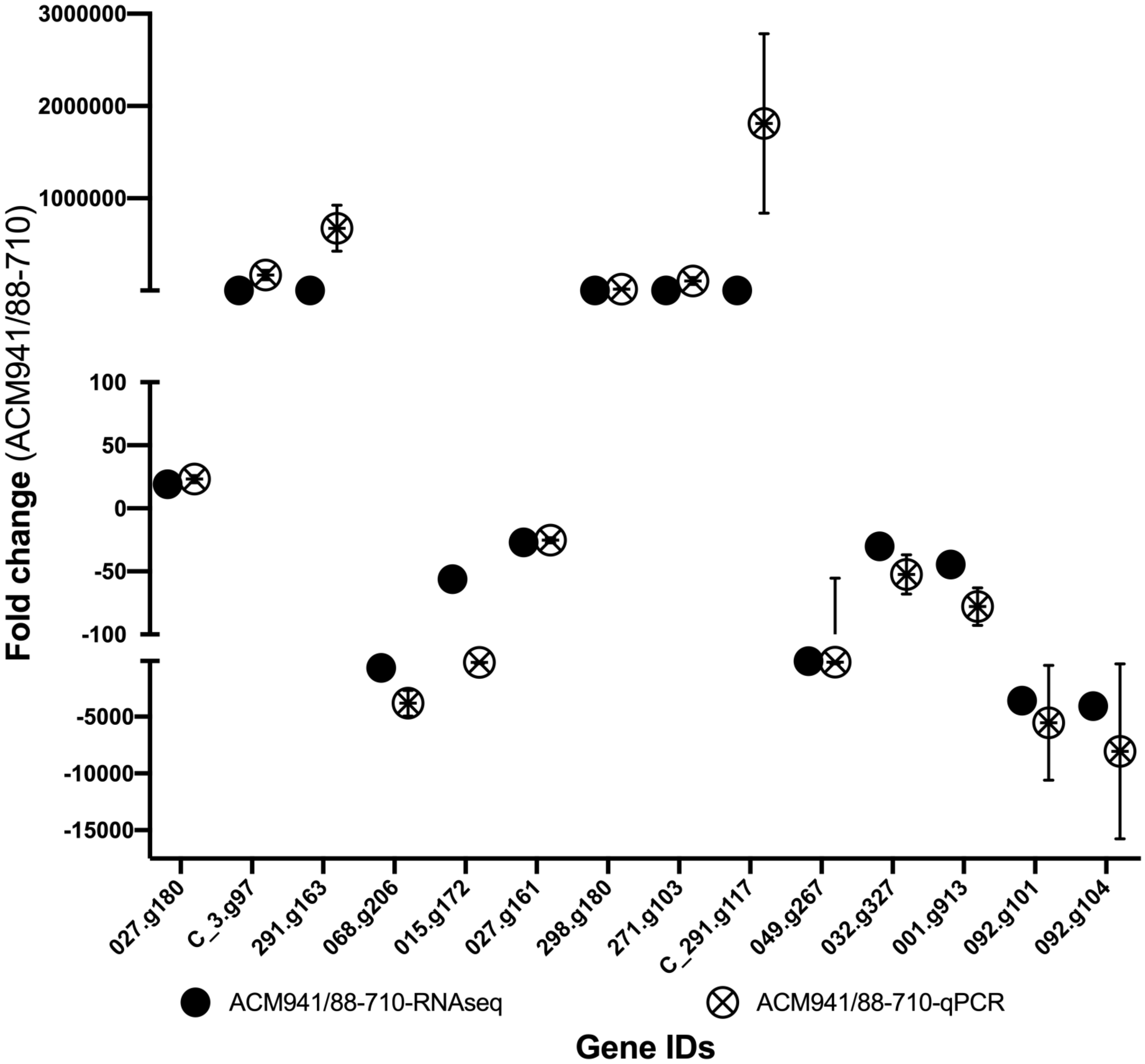

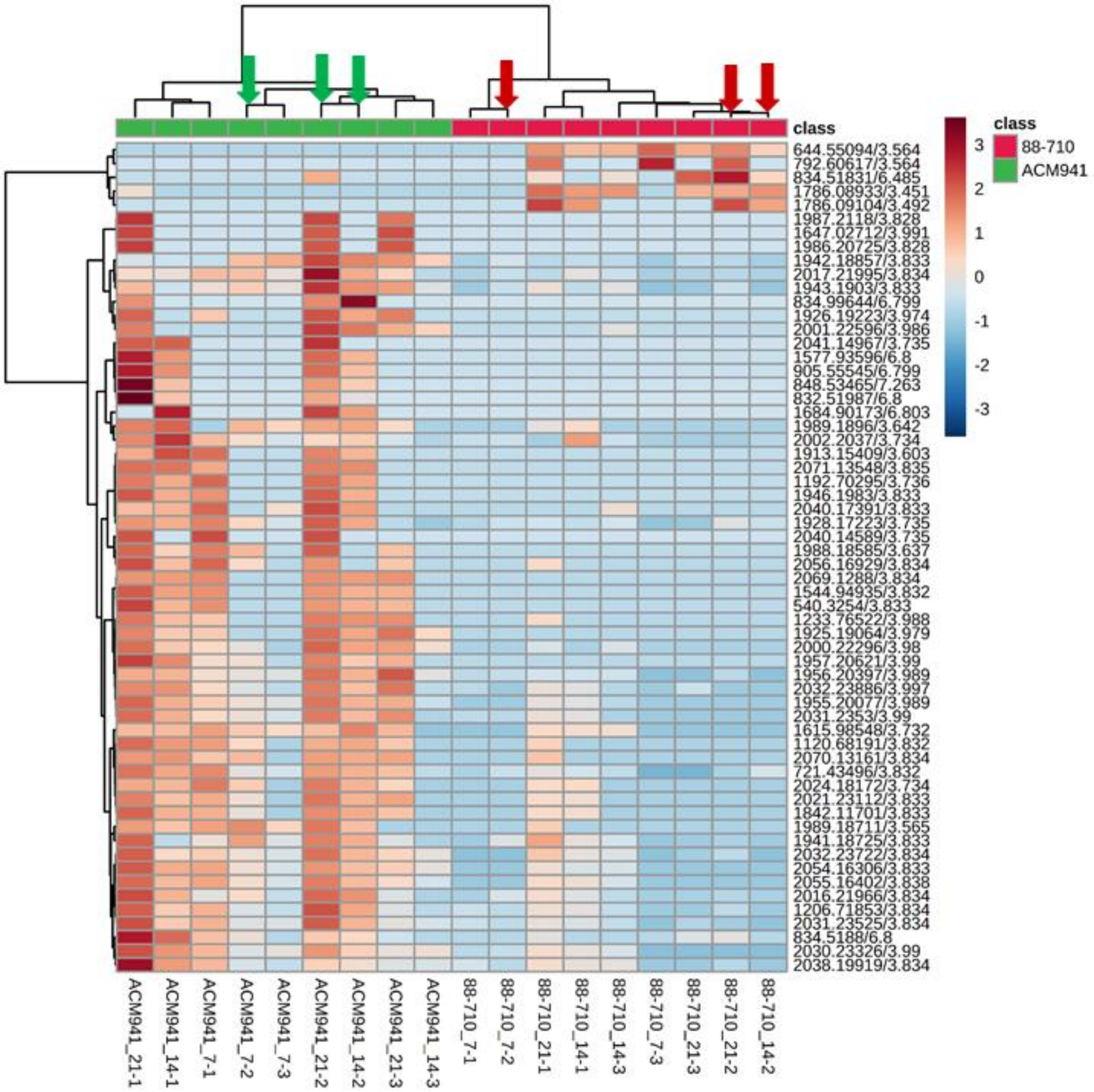

Fig. S5. Heatmap representation of the top 75 highly accumulated metabolites by *C. rosea* strains ACM941 and 88-710 after 7-, 14- and 21-days fermentation in Czapek Dox media. Metabolites are labelled based on mass/charge ratio and retention time (m/z/RT). For sample names, numbers after the hyphens represent replicate number.

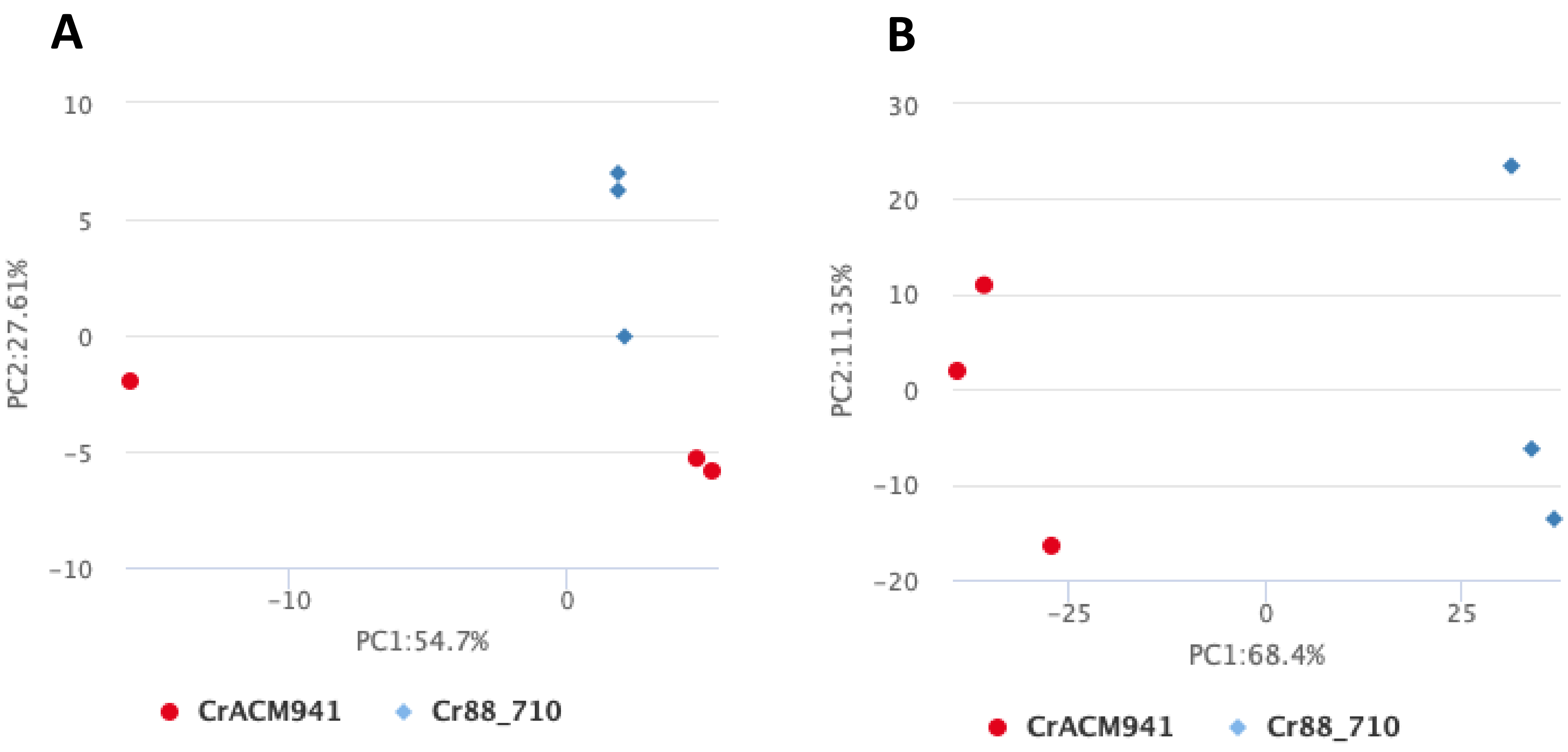

Fig. S6. Principal component analysis of metabolomics **(A)** and gene expression **(B)** data. Red coloured dots represent ACM941 and blue represent 88-710. Sampling was performed after 11 days of fermentation for transcriptomic and exometabolomics analysis.

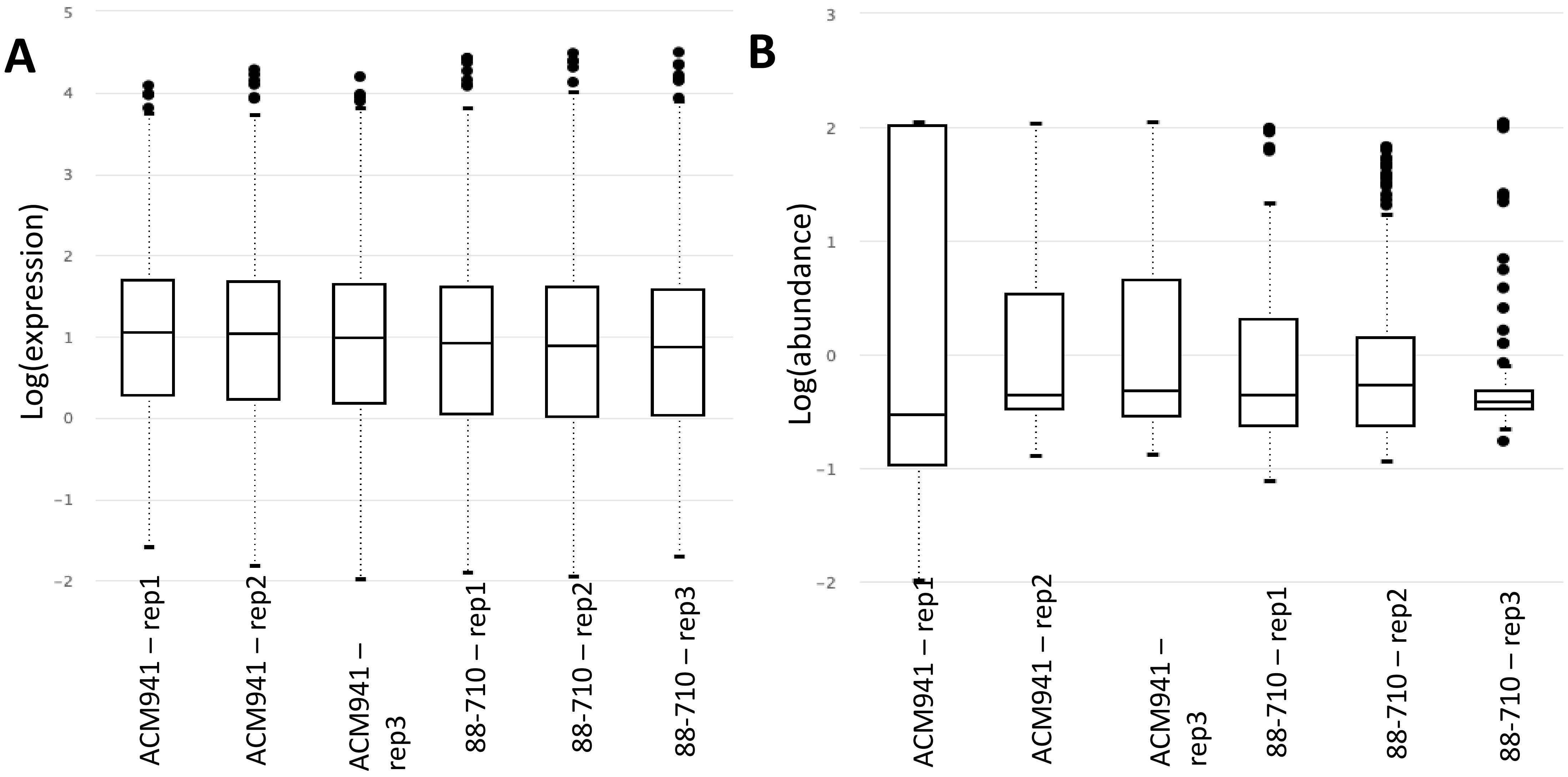

Fig. S7. Box plot representation of distribution of normalized and log2 transformed gene expression **(A)** and metabolomics **(B)** data.

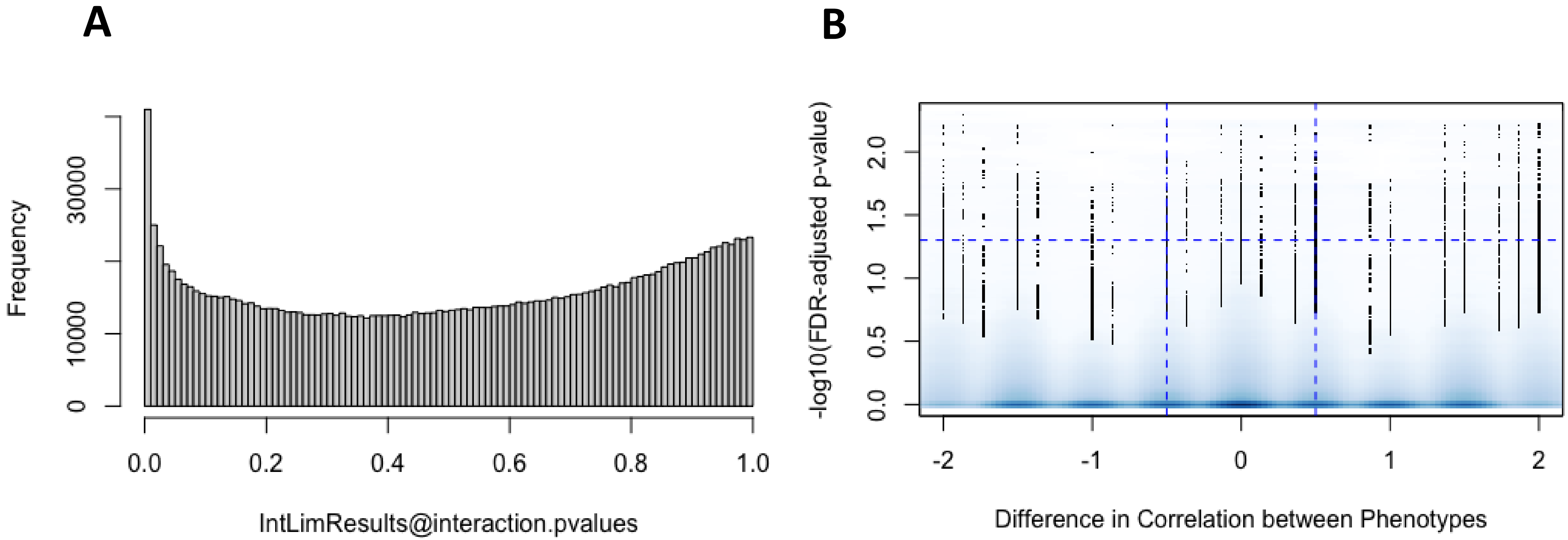

Fig. S8. IntLIM linear modeling gene-metabolite pairing. **A)** Histogram of Interaction p-values. All possible gene-metabolite pairs and extract FDR-adjusted interaction p -values < 0.05. **B)** A volcano plot depicting the absolute difference in correlation vs. – log10 (FDR-adjusted p-values < 0.01).

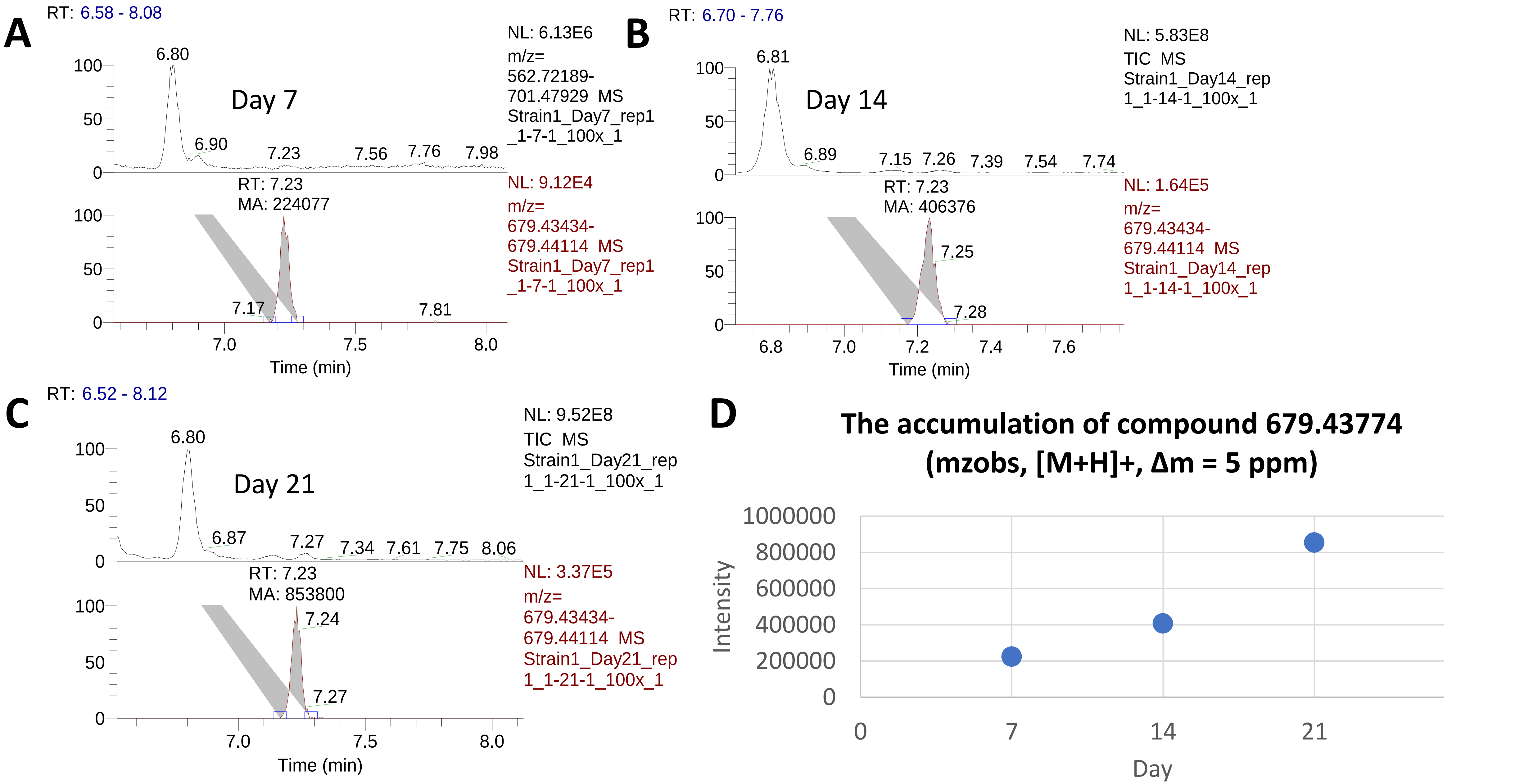

Fig. S9. Compound with mz_{obs} 679.43774 elutes at 7.23 minutes is consistent with observed. Extracted ion chromatogram at **A)** Day 7, **B)** Day 14 and **C)** Day 21 for mz_{obs} 679.43774 ($[M+H]^+$). **D)** The intensity of this metabolite was found to double every 7 days.

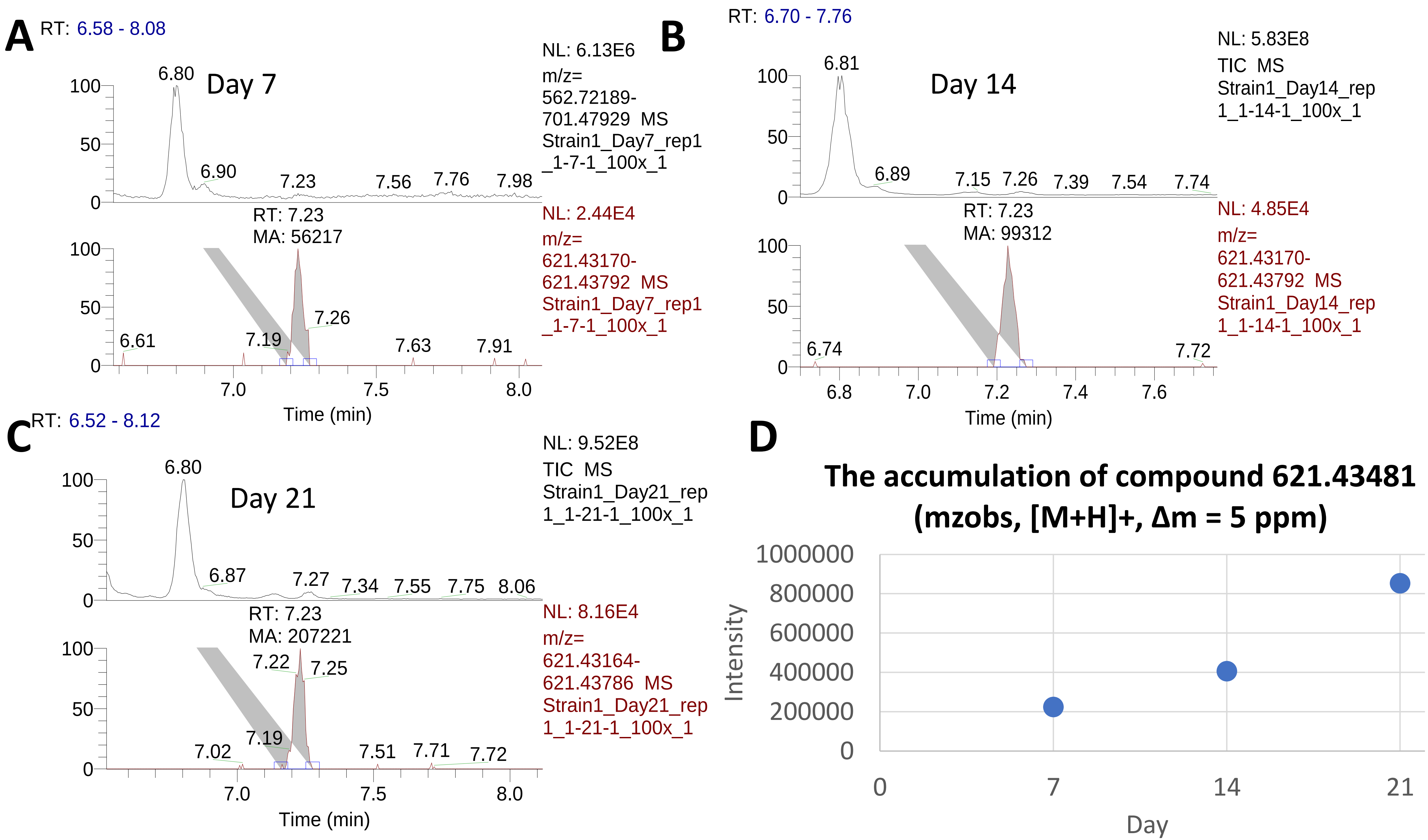

Fig. S10. Compound with mz_{obs} 621.43481 also elutes at 7.23 minutes. Extracted Ion chromatogram at **A)** Day 7, **B)** Day 14 and **C)** Day 21 for mz_{obs} 621.43481 ($[M+H]^+$). **D)** The intensity of this metabolite was also found to double every 7 days.
